# The Quantitative Methods Boot Camp: Teaching Quantitative Thinking and Computing Skills to Graduate Students in the Life Sciences

**DOI:** 10.1371/journal.pcbi.1004208

**Published:** 2015-04-16

**Authors:** Melanie I. Stefan, Johanna L. Gutlerner, Richard T. Born, Michael Springer

**Affiliations:** 1 Department of Neurobiology, Harvard Medical School, Boston, Massachusetts, United States of America; 2 Department of Biological Chemistry and Molecular Pharmacology, Harvard Medical School, Boston, Massachusetts, United States of America; 3 Department of Systems Biology, Harvard Medical School, Boston, Massachusetts, United States of America; University of British Columbia, Canada

## Abstract

The past decade has seen a rapid increase in the ability of biologists to collect large amounts of data. It is therefore vital that research biologists acquire the necessary skills during their training to visualize, analyze, and interpret such data. To begin to meet this need, we have developed a “boot camp” in quantitative methods for biology graduate students at Harvard Medical School. The goal of this short, intensive course is to enable students to use computational tools to visualize and analyze data, to strengthen their computational thinking skills, and to simulate and thus extend their intuition about the behavior of complex biological systems. The boot camp teaches basic programming using biological examples from statistics, image processing, and data analysis. This integrative approach to teaching programming and quantitative reasoning motivates students’ engagement by demonstrating the relevance of these skills to their work in life science laboratories. Students also have the opportunity to analyze their own data or explore a topic of interest in more detail. The class is taught with a mixture of short lectures, Socratic discussion, and in-class exercises. Students spend approximately 40% of their class time working through both short and long problems. A high instructor-to-student ratio allows students to get assistance or additional challenges when needed, thus enhancing the experience for students at all levels of mastery. Data collected from end-of-course surveys from the last five offerings of the course (between 2012 and 2014) show that students report high learning gains and feel that the course prepares them for solving quantitative and computational problems they will encounter in their research. We outline our course here which, together with the course materials freely available online under a Creative Commons License, should help to facilitate similar efforts by others.

This is part of the *PLOS Computational Biology* Education collection.

## Background

Modern biology increasingly requires computational and quantitative methods to collect, process, and analyze data, as well as to understand and predict the behavior of complex systems. Whereas throughout much of the 20th century computational and mathematical biology were niche disciplines, their methods are now becoming an integral part of the practice of biology across all fields [1]. It is therefore increasingly important that life scientists receive appropriate quantitative and computational training [2,3]. The importance of such training has been recognized at all levels from K–12 to continuing professional development [1,3–10]. Unfortunately, biologists’ competencies in computational and mathematical techniques often lag far behind the demands of the discipline [1,3,11].

To address this skill and knowledge deficit, doctoral training programs in biology need to offer computational and quantitative instruction that supports students’ research work and scholarship and sets them up for careers after graduate school. Biology majors have articulated their need for training in the skills of algorithmic thinking, problem solving, data analysis, and statistics [3]. Postgraduate education faces the additional challenge that incoming students vary widely in their background knowledge in these areas. Designing and offering instruction that accommodates such a diverse group of students is therefore a major challenge in graduate education within the biological and biomedical sciences.

In an attempt to address the need for quantitative and computational education at the graduate level, students are often directed to computer science or statistics courses. This strategy has several drawbacks. First, a typical programming or statistics course goes into more theoretical depth than many life scientists will need. Second, and more problematic, multiple courses are needed to obtain the basic quantitative and programming skills that are essential for solving real biological problems. This involves an extensive time commitment that most trainees cannot accommodate. In addition, typical programming and quantitative courses often teach concepts without linking them to possible applications in the life sciences, making it difficult for students to recognize their value in the context of their own research, and thereby missing an opportunity to motivate and engage these students.

A number of successful initiatives to increase quantitative and computational education for biologists have been launched in recent years, most of them at the undergraduate level. They often share common characteristics that promote their success: computational methods are taught in the context of biological examples [4,12,13], courses use active and hands-on learning methods [4,12,14,15], and quantitative instruction is coordinated across courses into integrated interdisciplinary curricula [13–16]. Here, we describe a course in quantitative methods offered to biology graduate students at Harvard Medical School that makes use of many of the approaches successful at the undergraduate level while creating a unique experience for graduate students.

The Quantitative Methods Boot Camp course (QMBC) is an approximately 50-hour, hands-on course that introduces students to the fundamentals of programming, statistics, and image and data analysis through the use of MATLAB [17]. Throughout the course, students apply the concepts they learn to examples from biology, including, if they choose, their own research data. In this paper, we present the course learning goals and the design and pedagogical methods we employ. Finally, we discuss data from the postcourse surveys from the last five offerings, which reveal that students enjoy the course overall, report high learning gains, and are inspired to use quantitative and computational methods in the future.

### Learning Goals and Objectives

QMBC is guided by a set of overarching learning goals that we want our students to achieve and retain in the long term. We broadly categorize these goals into three domains, “thinking,” “doing,” and “feeling.” This reflects our belief that developing practical programming skills (“doing”) is of limited use if one does not also develop both the ability to think about problems algorithmically (“thinking”) and a positive attitude towards computing (“feeling”).

“Thinking” goals include skills such as breaking a complex problem into simpler steps, re-casting questions in a way that makes them amenable to computational analysis, and developing strategies to validate a specific problem-solving approach.

We use the category of “doing” to describe the practical programming skills students need to implement their problem-solving strategies. The course introduces them to MATLAB [17] as a tool for achieving this, but nearly all of the skills and concepts are transferable to other computer programming languages.

In the domain of “feeling,” our goals focus on fostering a positive attitude towards quantitative and computational thinking. A major challenge in this arena is helping the students overcome their initial apprehension towards computer programming. By the end of the course, we want students to be comfortable with computational tools and quantitative reasoning, and to recognize their value in a biological research context. Throughout the course, we encourage students to experiment and play with the code and to follow their curiosity.

An overview of our goals in all three domains is given in Table 1.

**Table 1:** Learning goals for QMBC.

**Thinking**
Students will be able to
- recognize situations that call for computational methods
- conceptualize a problem so it becomes amenable to computational solution
- use simulations to build intuition about biological systems
- compare the outcome of simulations to real-world data
- formulate and test hypotheses
- understand a project as a collection of smaller parts
- plan steps needed to solve a problem
- think of ways to test the validity of a computational approach
**Doing**
Students will be able to
- import large datasets into MATLAB
- parse such datasets into appropriate computational structures
- visualize a dataset in multiple ways
- compute summary statistics
- use elements of programming to implement problem-solving strategies
- use trial and error to design a computational approach
- read and understand MATLAB documentation
- read and understand someone else’s code
- find and fix errors in a piece of code
- write a program to automatize data analysis
- document their code and use programming style in naming variables
**Feeling**
Students will
- appreciate the value of computational and quantitative approaches
- feel confident about approaching and solving a computational problem
- persevere when they find a problem difficult or do not immediately understand it
- recognize that successful coding can be fun as well as useful
- know when to ask for help and where to find support when needed
- be willing and ready to learn more
- evaluate the quality of computational and quantitative methods in scientific studies
- influence the work of others by setting examples of good practice in this domain

Learning goals for QMBC, categorized into the three domains of thinking, doing, and feeling.

The course consists of five full days of instruction: Days 1 and 2 provide an introduction to programming using MATLAB, Day 3 is devoted to approaches to probability and statistics, including common misconceptions and possible pitfalls [18], Day 4 to image analysis, and Day 5 to students’ own data or other special topics (Tables 2 and 3 and S5 Text). The topics covered in the course were chosen based on a combination of student interest, didactic benefit, and curricular need. Independent of their specific research field and topic, all our students will need to know about statistics and data analysis, both for their own projects and in order to understand the scientific literature. Likewise, data in many areas of biology comes in the form of images (often in large numbers), so most of our students will benefit from a knowledge of how to process images and extract information. To match changing demand over the past few years we have introduced, and plan to continue to develop, more bioinformatics exercises. Because any concept can be understood at different levels [19,20], and students start the journey towards more sophisticated understanding at different entry points and progress at different rates [21], being mindful of these individual differences is key to offering a course that benefits all students.

**Table 2:** Overview of course Days 1–3.

Topic	Exercise/ Examples	Biological problem
**Day 1**		
Getting Started		
Variables	Creating variables; basic operations on variables	
Arrays	Indexing, storing, retrieving, and elementary operations	Image visualization
Built-in Functions	Summary statistics	
Data visualization	Histograms, color maps, and plots	
INTEGRATION	Summary statistics and plotting to characterize an unknown dataset	Mystery ‘microarray’ dataset
Arrays II	Cropping and subsampling	Image manipulation
Conditional statements	Logical operations on arrays (<, >, = =)	
INTEGRATION	Normalize and modify an image with built-in functions and logical operators	Image manipulation and visualization
INTEGRATION	**Compare single cell reporter expression from images of co-cultured wild-type and mutant cells**	
**Day 2**		
Review of Day 1		
Functions	Inputs, outputs, scope, and naming	
Functions	Convert script from Day 1 into a function	Image normalization and visualization
Loops	for	
Conditional statements	if, elseif, else, while	
INTEGRATION	96-well plate growth curve data	
Strings	Data type conversion and basic pattern matching	Basic bioinformatics (find a ‘motif’)
Cell arrays	Dealing with mixed data types	Data plus metadata
INTEGRATION	**Yeast cells: Protein expression changes and cell growth over time—image series**	
**Day 3**		
Binomial distribution, null hypothesis, p-value	Binomial rat—simulation	Choice behavior in animals
Bootstrapping methods	2-sample neuron comparison—resampling	Morphological characterization of neurons
False positive statistics	“researcher degrees of freedom” and multiple hypothesis testing	Neuronal data—simulation

Summary of the topics covered in Days 1–3 of the course, the examples and exercises, and the biological motivation.

**Table 3:** Overview of course Days 4 and 5.

Topic	Exercise/ Examples	Biological problem
**Day 4**		
Visualizing and scaling images	Images with varying dynamic ranges	
Segmentation versus quantitation	Counting and characterizing cells	
Filtering	Understanding filters, visualization of their effects on different images, combining filters	
Edge detection	Segmentation by finding boundaries of cells	
Morphological operations	Quantitation and segmentation of an image with uneven illumination	
**Day 5**		
Loading and parsing data	Uploading and parsing an RNA sequencing experiment	
INTEGRATION	**What is the effect of KCl on neuronal gene expression?—RNA sequencing time courses with replicates**	
**Options**	(Advanced topics and integration):	
Bring your own data		
Bootstrapping	Neural tuning curves	
Principal component analysis	Spike sorting or Calcium imaging in zebrafish	
Biological image processing	Image filters used by biological vision systems	
Quantitative trait loci	Raw sequencing data → enriched alleles: identifying causative loci	
Pattern matching	Identifying over- and underrepresented motifs in a genome	
Biochemical/signaling models	Introduction to simbiology and simple models	

Summary of the topics covered on Days 4 and 5 of the course, the examples and exercise, and the biological motivation.

For each day, we have defined a set of learning goals and objectives (S1 Text). Students complete exercises that help them attain those learning goals. Wherever possible, we draw on examples from biological research, as illustrated in the following example from the first day.

### Comparing Galactose Metabolism in Two Yeast Strains

The first day of the course serves to introduce students to the MATLAB environment and to teach them to analyze single data points, work with arrays of data points, and visualize and explore data. At the end of the day, students attempt an hour-long exercise that serves to synthesize these concepts and to apply them to a problem commonly encountered in biological research: the analysis of images from a microscope.

The biological question for this exercise concerns the ability of two different yeast strains to metabolize galactose. The strains are distinguished by the expression of different fluorescent proteins (red and blue), and galactose production is read out by a third (yellow) fluorescent protein (YFP). The students are given three images corresponding to the three different fluorescent channels. They must first select cells according to which yeast strain (red or blue) they belong, and then measure YFP levels for each strain and compare them.

The experiment is first described by the instructor, and there is an in-class discussion that helps students understand the problem and plan the steps needed in solving it. Students then work at their own pace, either individually or with a partner. They can tackle the problem creatively, or follow a set of written guidelines (S2 Text, page 1). Instructors and teaching assistants walk around the classroom to answer questions and offer feedback. This is a purposefully difficult exercise, and we do not expect most students to complete it within class. The completion of the exercise is one of the homework assignments for Day 1.

Working through the yeast metabolism exercise, students have to load and visualize images, work with data arrays, extract information from them and compute summary statistics, all of which are learning goals for Day 1. The exercise also reinforces some of our overarching course goals in the “thinking,” “doing,” and “feeling” domains, such as planning the steps needed to solve a problem, using trial and error to solve a computational problem, and persevering through a complex problem.

A more detailed description of the context, delivery, and learning goals for this exercise is provided in S2 Text (page 2 onward).

A similar discussion of an exercise from the Statistics module of the course is presented in S3 Text (text of the exercise and detailed description of context, delivery, and learning goals), and S1 Code (MATLAB code). In this exercise, known as “Rattus binomialis,” students are presented with data from a behavioral experiment in which a rat must identify which of two odors was presented on a given trial [22]. They are asked to calculate how likely it is that the rat guessed correctly on 31 (or more) trials out of a total of 50. They calculate a p-value using Monte Carlo simulations [23], which reinforces key programming skills such as for-loops and logical indexing, as well as the use of MATLAB functions for generating random numbers.

### Course Structure and Pedagogy

QMBC is offered twice a year in order to accommodate both beginning and advanced graduate students. Incoming graduate students can take the course before the start of fall semester and thus be exposed to quantitative and computational training early in graduate school. Students who realize the need for such training later in their studies can take the spring offering of the course.

The course is co-taught by two faculty course directors, a team of teaching assistants, and a curriculum fellow. Curriculum fellows are PhD level scientists who support curriculum design, improvement, and innovation [24]. Teaching assistants are recruited from graduate students in our departments who use MATLAB in their research work. This means that they are both proficient in MATLAB, and also that they can speak to the applications of computational tools in their scientific area of interest, thus serving as peer role models to our students. Many of our teaching assistants have taken QMBC before, and are thus familiar with the course structure and content. TAs prepare for the course by going through the course material online and attending a one-hour training session prior to the course.

Because our teaching approach requires hands-on exercises and individualized support, we have found a ratio of approximately one instructor for every seven students to be ideal given the resources available to us. However, we have found that certain parts of the course require more intensive assistance than others, and this knowledge could be used to more effectively allocate a smaller number of TAs.

The course is structured as a two-week intensive “boot camp” in which five full, mandatory days alternate with four optional half-day sessions (16 additional contact hours) that allow students to reinforce core skills and concepts. Student enrollment for the past five offerings is shown in Table 4.

**Table 4:** Student enrollment in QMBC.

Spring 2012	Summer 2012	Spring 2013	Summer 2013	Spring 2014
74	94	37	74	44

Student enrollment in QMBC, Spring 2012 to Spring 2014

Instruction in QMBC is focused on practice, with short lecture segments interspersed with self-paced problems like the one discussed above to allow students to implement, reinforce, and synthesize new content. All concepts in QMBC are taught using MATLAB [17]. While students are solving these problems, instructors and teaching assistants walk around the room to answer questions, discuss possible solutions, and provide feedback. In addition, we encourage students to work together in small groups.

The interaction with instructors provides students and instructors with continuous feedback on their work. To further facilitate this type of formative assessment, lecture segment and practice modules are supported by Learning Catalytics, an online platform through which students can answer questions in real time, provide feedback on their understanding of the course materials, and complete exercises (S4 Text) [25]. Students can find course schedules, exercises, and other information on our course website at http://springerlab.org/qmbc/. Course content and exercises can also be accessed on GitHub under https://github.com/MelanieIStefan/QMBC. The materials are licensed under a Creative Commons Attribution-NonCommercial-ShareAlike 4.0 International License (http://creativecommons.org/licenses/by-nc-sa/4.0/). The course is graded on a pass/fail scale and students are assigned a passing grade based on attendance at all full-day sessions and completion of all in-class and homework exercises.

### Course Evaluation

**Fig 1:**
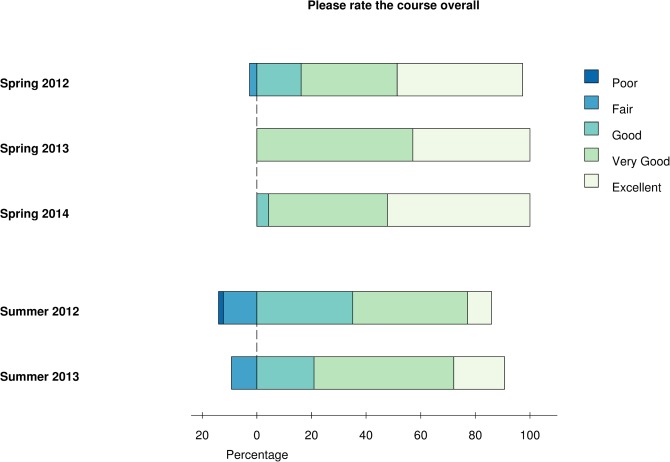
Overall course experience. Students were asked after each course to rate their overall experience on a five-point scale (Poor, Fair, Good, Very Good, or Excellent). Diverging stacked bars are centered between Fair and Good. To allow comparison between different course offerings, data for each year was normalized by the total number of respondents. Spring 2012: *n* = 37, Spring 2013: *n* = 21, Spring 2014: *n* = 24, Summer 2012: *n* = 57, Summer 2013: *n* = 43.

We ask students to complete postcourse surveys after every offering. Results from the last five offerings (Spring 2012 to Spring 2014; see S9 for details), reveal that students report a positive course experience overall (Fig 1), with a large majority of respondents rating the course as “good,” “very good,” or “excellent.” Overall ratings are better for spring courses (97%, 100%, and 100% of ratings “good” or better) than for summer courses (86% and 91%). Spring courses are offered to upper-division graduate students and a few postdoctoral researchers, while summer courses are offered to incoming graduate students (and participation is highly recommended by some of the graduate programs). This results in the summer offering usually having a larger class size. In addition, students who elect to take the class in spring are often motivated by a concrete research problem they want to solve and are typically beginners or near-beginners at programming. In summary, our spring classes tend to be smaller, more motivated, and more homogeneous, all of which might contribute to increased student satisfaction. In both spring and summer offerings, we see a general trend of improvement in students’ ratings over the years the course has been offered, reflecting the success of iterative improvements in course structure, material, and delivery (S5 Text).

**Fig 2:**
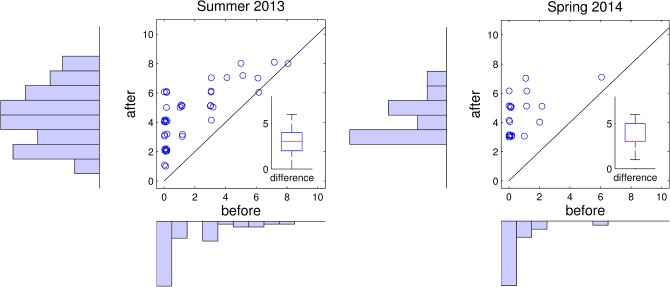
Increase in self-reported MATLAB programming skills. In the postcourse survey, students were asked: “Rate your ability to program in MATLAB before the course,” and “Rate your ability to program in MATLAB after the course.” Answers were given on a scale from 0 (novice) to 11 (expert). Upper panel: Summer 2013, lower panel: Spring 2014. Scatter plot: Each student is represented by a circle. The diagonal represents no improvement in skill. Insert: Increase in self-reported skill (after-before). Summer 2013: *n* = 43, Spring 2014: *n* = 24.

**Fig 3:**
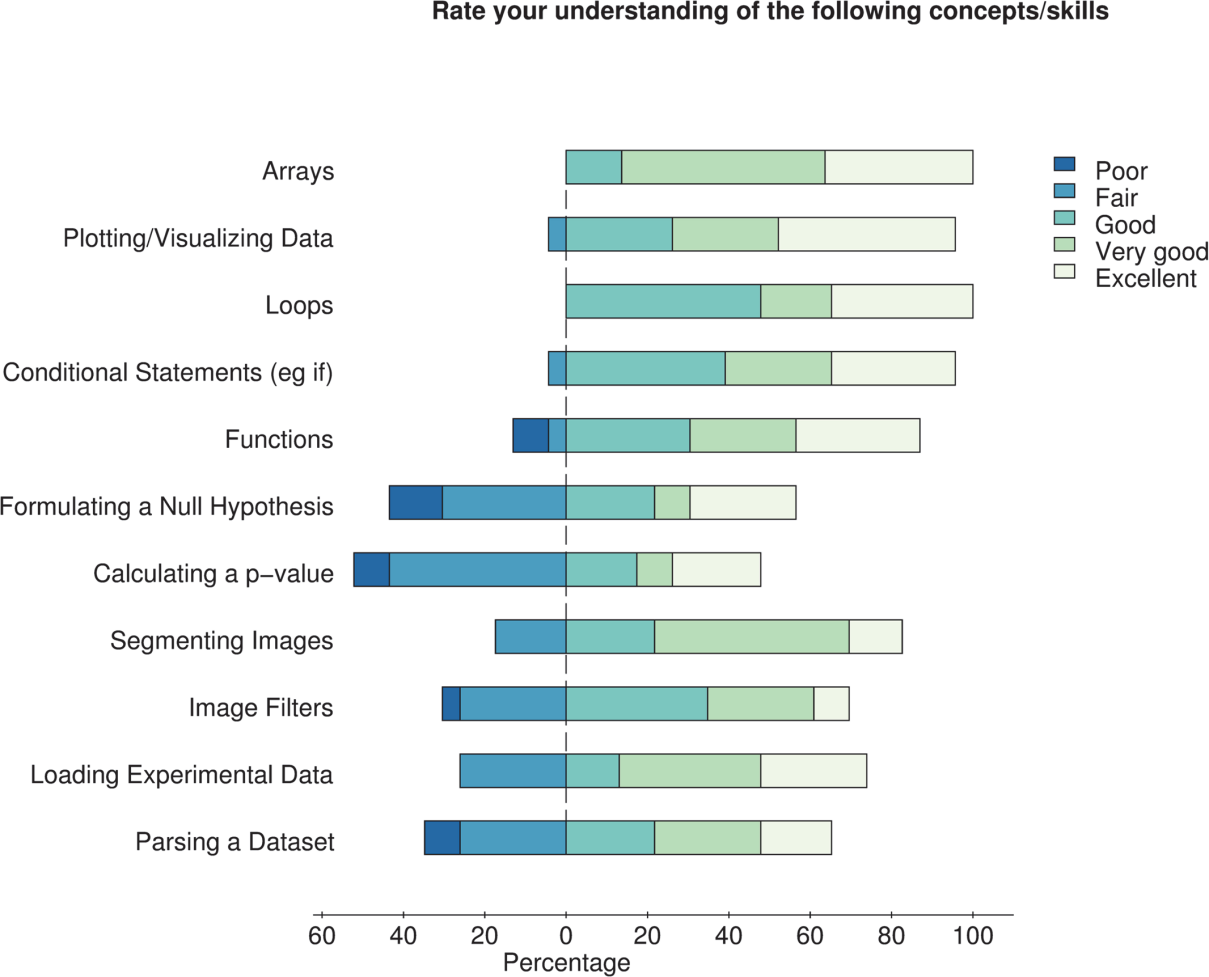
Self-assessed understanding of concepts and skills. Data shown is for the Spring 2014 offering of the course. Students were asked to rate their understanding of specific skills on a five-point scale (Poor to Excellent, as above). Skills are listed in the order in which they are introduced at QMBC. *n* = 24.

In terms of learning outcomes, students show significant gains in their self-assessed programming ability (Fig 2 and S5 Text). They report a good understanding of programming skills taught early in the course, and find some of the concepts around statistics most challenging (Fig 3 and S5 Text).

**Fig 4:**
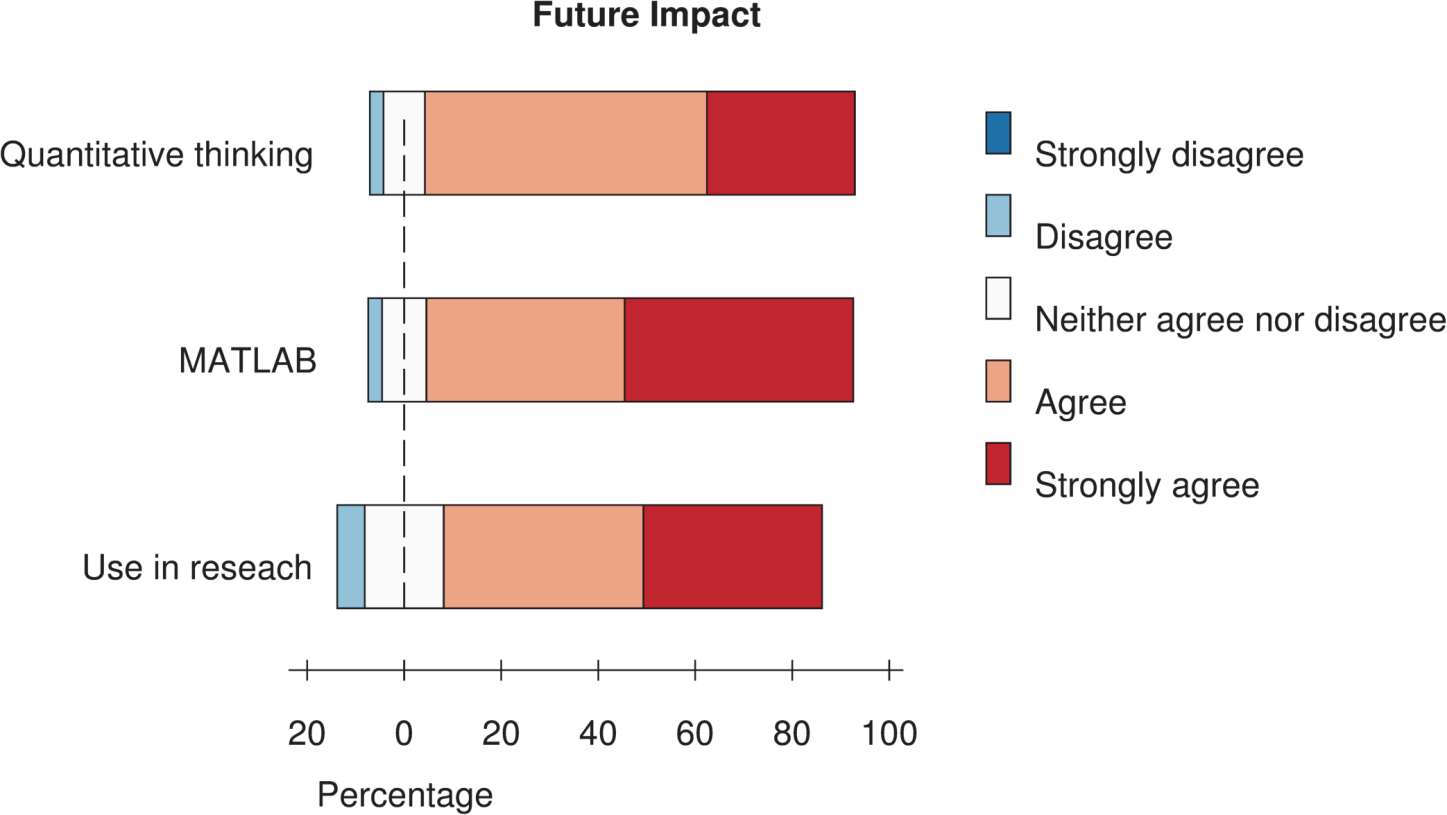
Future impact of the course. Students were asked to rate their agreement with the following three questions: “This course provided a practical base and starting point for using MATLAB in my own work,” “The workshop provided me with a practical base/starting point for analyzing quantitative problems,” and “This course has increased the likelihood I will use quantitative methods in my research.” Rating was on a five-point scale (Strongly disagree to strongly agree). Data shown are pooled responses from the last four offerings of the course (Summer 2012 to Spring 2014). *n* = 141.

The postcourse survey also asked students to evaluate the potential impact of the course on their research and scholarship. This allows us to see whether we are likely to have met our longer-term learning goals and to gauge students’ attitudes towards using computational tools and quantitative reasoning in their future careers. Survey responses indicate that students feel they have acquired practical and cognitive skills that prepare them to use quantitative and computational methods in their work and scholarship, recognize the value of computational and quantitative approaches, feel confident using them, and would encourage others to learn more about them (Fig 4 and S5 Text).

### Continuing Support

After completion of QMBC, students have regular opportunities to maintain and develop their quantitative skills. Computational modules are increasingly included across the curriculum in the Harvard Medical School’s Programs in Graduate Education. Students also have the opportunity to take computationally oriented “nanocourses” offered at Harvard Medical School [26]. In addition, we have initiated a weekly data club/help desk session where students can bring quantitative and computational questions that have arisen in their research. Thus QMBC is embedded within a wider quantitative curriculum for graduate students. We hope that this will be part of a community effort to develop material that can be shared between courses across universities.

Students wanting to improve their quantitative and computational skills now have unprecedented access to training materials and courses online, including through dedicated learning platforms such as codecademy (http://www.codecademy.com/about) or the Software Carpentry project (http://software-carpentry.org), and through a wide range of Massive Open Online Courses [7,8]. With our two-week boot camp, we want to teach basic concepts, increase students’ confidence and awaken their curiosity and willingness to learn more, so that they can benefit from other educational opportunities, both on and off campus. Ideally, QMBC will be the beginning of a lifelong learning journey in this particular field.

## Conclusion

QMBC offers targeted instruction in quantitative and computational skills for life science graduate students at Harvard Medical School. The course benefits from a hands-on approach to programming, ample opportunity for practice in dedicated sessions, and structured exercises that appeal to beginners and advanced students alike. We are fortunate enough to be able to run the course with a high instructor-to-student ratio (about 1:7), which we feel is helpful, but not indispensable for reaching our course goals. Evidence from postcourse surveys indicates that the course helps students to develop their quantitative problem-solving skills, increase their programming ability, and develop a positive attitude towards quantitative thinking. While our course is geared for graduate level biologists, we believe the approaches we take are widely applicable both to other graduate disciplines and at the undergraduate level. Additionally, we believe the content and approaches we use here could be adapted to a semester-long course that employs classroom time for independent and group work on programming exercises.

As described here, this course helps to lower the activation barrier for students to engage with computational methods and has increased the number of students using computation approaches in our graduate programs. However, the course alone is not a panacea. To be truly successful, computational problems and approaches must be integrated throughout the graduate curriculum that follows the boot camp, so that students continue to gain proficiency and expertise throughout their graduate career. If we can succeed in this effort, our graduate students will be well positioned to grapple with the experimental questions of the 21st century.

## Supporting Information

S1 Code Solution to the Rattus binomialis exercise. (ZIP)

S1 Text Daily learning goals and objectives for QMBC. (ZIP)

S2 Text Text of the yeast metabolism exercise and detailed description of the exercise. (PDF)

S3 Text Text of the Rattus binomialis exercise and detailed description of the exercise. (PDF)

S4 Text Why we use Learning Catalytics. (PDF)

S5 Text Details on course assessment and students’ rating of QMBC. Includes Figures S1–S20.
(PDF)
