# Supplementary material for: The Quantitative Methods Boot Camp: Teaching Quantitative Thinking and Computing Skills to Graduate Students in the Life Sciences
Source: PLoS Comput Biol. 2015 Apr 16;11(4):e1004208. doi: 10.1371/journal.pcbi.1004208 (PMC4399943; doi:10.1371/journal.pcbi.1004208)
Supplement: S1 Text — (ZIP) [file pcbi.1004208.s002.zip › S1.html]

Quantitative Methods Boot Camp


All +
All -

Quantitative Methods Boot Camp

- + -
  Day 1: Programming Part 1
  - + -
    Feel comfortable using a quantitative tool
    - Navigate the MATLAB workspace
    - Define, view, and clear variables
    - Use help functions
  - + -
    Analyze a single measurement
    - Use operations like addition and multiplications on variables
    - Use built-in functions (pi, sin, mean, median, ...)
    - Transform variables (rounding, taking logarithms, absolute values, ...)
  - + -
    Analyze multiple measurements
    - Define an array
    - Load an array into MATLAB
    - Find and address entries within an array
    - Perform array operations
    - Access information about an array
    - Define and use special arrays (ones, zeros, eye)
  - + -
    Visualize data
    - Show images in MATLAB
    - Create a plot or histogram
    - Edit properties of an image
  - + -
    Explore a dataset
    - Compare two datasets
    - Compute descriptive statistics
    - Explore a dataset by using different visualization tools
- + -
  Day 2: Programming Part 2
  - + -
    Work with multiple datasets
    - Use loops to perform a set of actions repeatedly
    - Use if statements to check whether a condition has been fulfilled
    - Replace some loops with appropriate array operations
  - + -
    Streamline data analysis
    - Write and run scripts
    - Write and run functions
    - Test and debug scripts and functions
  - + -
    Solve a problem by breaking it into small steps
    - Use pseudocode to outline the steps of a solution
    - Document parts of a script
- + -
  Day 3: Statistics
  - + -
    Visualize and summarize data
    - Plot experimental data and outcomes of simulations
    - Compute summary statistics from a dataset
  - + -
    Use simulations to build intuition about statistical concepts
    - Use computer simulations to build intuition about random variables
    - Simulate different kinds of random distributions
    - Simulate sampling distributions
    - Use computer simulations to inform experimental design
  - + -
    Use simulations for hypothesis testing
    - Articulate and encode the Null Hypothesis
    - Generate a simulated distribution under the Null Hypothesis
    - Compare simulated results to data and obtain a p value
    - Accept or reject a hypothesis based on the outcome of a simulation experiment
    - Compare simulation-based approaches to standard hypothesis tests
- + -
  Day 4: Image analysis
  - + -
    Load and visualize images
    - Load images
    - Perform simple image manipulations (crop, normalize, filter...)
    - Visualize single images
    - Visualize a sequence of images as a movie
  - + -
    Find objects or regions of interest
    - Use thresholding to identify regions of interest
    - Segment an image by using a binary mask
  - + -
    Measure features of an image or object
    - Access features of images (e.g. size, brightness)
    - Quantify properties of selected regions or objects
    - Compare images with respect to specific features or properties
  - + -
    Create quality-controlled image analysis workflows
    - Test automated image analysis methods by comparing them to manual analysis
    - Plan an image analysis and quality control workflow
    - Scale automated image analysis by using loops and functions
- + -
  Day 5: Selected topics
  - + -
    Apply concepts to one's area of interest
    - Import and parse large datasets
    - Solve a problem from one's area of interest
    - Create and implement a data analysis workflow for one's own research data
    - Desing and plan experiments
