# Supplementary material for: The Quantitative Methods Boot Camp: Teaching Quantitative Thinking and Computing Skills to Graduate Students in the Life Sciences
Source: PLoS Comput Biol. 2015 Apr 16;11(4):e1004208. doi: 10.1371/journal.pcbi.1004208 (PMC4399943; doi:10.1371/journal.pcbi.1004208)
Supplement: S2 Text — (PDF) [file pcbi.1004208.s003.pdf]

Rattus binomialis

MATLAB concepts covered:

1. 'rand'
2. simulations for answering questions of probability
3. 'for' loops
4. indexing arrays
5. 'tic' and 'toc' to time code execution
6. binomial distribution
7. pdf and cdf

The file BinoRat.m contains one solution to this exercise. We refer to it at several points in this document, but we will only make it available to you after you have completed the exercise, so you can compare your solution to it. In the meantime, you can access http://www.springerlab.org/qmbc/hints.php or the bottom of this document for hints.

A rat is performing a "2-alternative forced choice" (2AFC) task in which it must identify an odor presented at a central port. If it detects odor 'A' it should choose the right-hand port for a reward; if it detects odor 'B' is should choose the other port.

See movie: uchida-mainen-rat_odor_discrimination.mov

The rat in the movie clip is highly trained and gets it right almost every time. But if we go back in time to the early training period, we would find that the rat seems to get it wrong as often as he gets it right. So you decide to do a test and keep track of his correct rate for a block of 50 trials. After 50 trials, we see that the rat has gotten 31 trials correct (19 trials wrong) for an average of 62% correct. **You want to know if the rat has learned the task or if he is still guessing.**

Let's say that we know nothing about probability theory or the binomial distribution, so we will just use the brute force power of a simple simulation.

What is the null hypothesis?

Since there are only two possible answers, this corresponds to a correct probability of 0.5, which is identical to the rate at which tossing a "fair" coin should produce a "head" (or a "tail"). So our previous question can be put as: **how often would one expect to get 31 (or more) heads out of 50 coin tosses**?

Why is the parenthetical "or more" added to the previous sentence?

Well, we're not really interested in the probability of the rat getting *exactly* 31 of 50 trials correct. We're looking for conditions under which we would *reject* the null hypothesis of guessing. So we would clearly count any performance that was better than 31 as well. To answer with a real coin would take a long time, but computers are fast, so we can let the computer do the flipping. How?

Step 1: Write a single line of MATLAB code to simulate a coin toss. It should return a '1' for heads and a '0' for tails.

How many ways can you think of to do this?

See Hint 1 below for help
Also see Step 1 in BinoRat.m

To address our particular question, we need to find the number of occurrences of tails in runs of 50 tosses.

How might we do this?

Step 2: Write a 'for' loop to simulate 50 coin tosses and store the results in an array called 'tosses'.

We want to know *how often* a certain number of tails is likely to occur in a run of 50 tosses, so we need to repeat the coin flip 50 times. Then we can ask whether, in those 50 tosses, we observed 31 or more tails. One way to solve this problem is by writing a ‘for’ loop.

What is a ‘for’ loop?

See Hint 2 below for help
Also see Step 2 in BinoRat.m

Let’s start by initializing some variables.

What types of variables will we need for this simulation?

Thus far, you’ve learned how to "grow" arrays within 'for' loops, but it's better form to have your arrays pre-declared, provided it's predictable in advance how many values you will be generating. In this case it is, so declare variables to hold the results of your simulations:

```
tosses = zeros(n,1); % results of individual coin tosses on one simulation
```

Why did we initialize this variable with zeros?

So, now we can write the ‘for’ loop to simulate 50 coin flips.

See Hint 2a below for help
Also see Step 2 in BinoRat.m

Step 3: Code a pair of nested 'for' loops that will do *nsim* simulations of 50 coin tosses and store the resulting number of heads on each simulation in *results*.

Step 2 had us simulate flipping a coin 50 times. But we want to flip a coin 50 times *lots of times* so that we can generate an understanding of how likely we are to turn up 31 tails.

How can we simulate 50 coin flips 1000 times?

See Hint 3 below for help
Also see Step 3 in BinoRat.m

What additional variables will we need?

To run our 1000 simulations of 50 coin flips each, we will write a nested for loop. Now we can ask how often a given result (31 or more tails) happens in 1000 simulations.

See Hint 3a below for help
Also see Step 3 in BinoRat.m

If you run your nested for loops again, do you get the same answer? How can you get a progressively "better" answer?

Step 4: Write a script that will do the same thing without 'for' loops.

Because MATLAB is a vector-based language, we can often do things much more efficiently using indexing and operators (such as '>') that work across entire arrays or functions that work along a specific dimension, such as down columns.

How might you solve this problem without relying on for loops?

See Hint 4 below for help
Also see Step 4 in BinoRat.m

This code will be much more satisfying code (fewer lines!), and it will execute much more quickly. You can test the speed using MATLAB's 'tic' and 'toc' functions. You execute a 'tic' at the beginning of your code, and then a 'toc' at the end, and you'll get the amount of elapsed time. Try this for your loops in Step 3 and compare it to the code in Step 4.

See Hint 4a below for help
Also see Step 4 in BinoRat.m

Step 5: Using a built in binomial function, write code that will give the probability of getting 5 or fewer heads in 10 tosses.

You might vaguely recall that events like coin tosses, where there are only two possible outcomes, are described by the binomial distribution[1]. Based on your knowledge of MATLAB, you might expect that there is an existing function that will make our task easier. Remembering only this key word, 'binomial', find some useful sounding functions.

Using one of these functions, what is the probability of getting (exactly) 5 heads from 10 tosses? Is there a single built-in function that can do this for us?

See Hint 5 below for help

Also see Step 5 in BinoRat.m

What are cumulative distribution functions (CDFs) and probability density functions (PDFs)?[2] Which function would be appropriate to use to determine the probability of getting 5 or fewer heads from ten tosses?

Step 6: Using 'binocdf', calculate the probability that the rat gets it right 31 times or more by just guessing.

Finally, let's return to our rat question. Remember, we want to know the probability of getting 31 tails or more out of 50 tosses.

How can we use binopdf to solve this question?
How can we use binocdf to solve this question?

See Hint 6 below for help
Also see Step 6 in BinoRat.m

OK, so what do we think? Is the rat "getting it" or is he just guessing? How many would he need to get right (out of 50) before you believed it?

How can we use the above functions to answer this?

EXTRA Step 7: Visualize the results from your simulation

Use the array named 'results' from Step 3 and plot its values.
Now, calculate the mean, standard deviation and standard error of 'results'. How do these values change as a function of the number of simulations?
Remake a histogram plot of 'results' but change the Y-axis values to percent occurrence rather than count number.

EXTRA Step 8: What if the distribution we cared about wasn't a binomial?

What other functions does Matlab make available? Inspect how they are distinct.
Find the relationship between a poisson distribution's mean and its variance.
Is there an easy way to generate random numbers from non-uniform distributions? Exponential? Normal? Plot them and compare them to the output of rand.
When would these other distribution types be useful?

EXTRA Step 9: Compute the 95% upper and lower confidence bounds for:

a) 30 correct out of 50
b) 60 of 100
c) 300 of 500
Is there a MATLAB function that does this auto-magically?

Footnotes

[1] The **binomial distribution** is the discrete probability distribution of the number of successes in a sequence of *n* independent yes/no experiments, each of which yields success with probability *p*.

[2] In probability theory, a **probability density function** (**pdf**), or **density** of a continuous random variable is a function that describes the relative likelihood for this random variable to occur at a given point. The probability for the random variable to fall within a particular region is given by the integral of this variable's density over the region. The probability density function is nonnegative everywhere, and its integral over the entire space is equal to one.

Likewise, the **cumulative distribution function** (**cdf**), describes the probability that a real-valued random variable *X* with a given probability distribution will be found at a value *less than or equal to x*. Intuitively, it is the "*area so far*" function of the probability distribution. Because the area under the pdf must sum to one, we can get the *"area beyond"* as: 1 – cdf(x).

Hints

Hint 1

Type `help rand`. How could you use the rand function to output zeros and ones only?

Hint 2

A for loop repeats a statement a specific number of times. Remember, a for loop is completed by an `end` statement.

Hint 2a

Once you have initialized the variables you need, you can use a for loop and the random coin toss function you used in step 1 to simulate a total of 50 coin tosses. The number of correct guesses will correspond to the number of times you get 1 in your coin toss. how can you keep track of that?

Hint 3

You have already coded a for loop that simulates 50 coin tosses. You can repeat this 1000 times by creating another for loop around this one. At the end of the series of 1000 experiments, you will probably want to have an array of length 1000 storing the result of each experiment. What exactly is the outcome (for each round of 50 coin tosses) you want to keep track of?

Hint 3a

You have encountered methods of comparing two numbers earlier (e.g. <, ==, >=). Use those to find out which of the simulations did as well as or better than your actual rat. How can you convert this number into a probability?

Hint 4

The key here is understanding that going through a loop *n* times is the same thing as performing the same operation or set of operations on all the elements of a vector of length n. For instance, the following two are equivalent

Code 1:

```
randomNumbers=[];
for i=1:5
    randomNumbers(end+1)=10*rand(1,1);
end
```

Code 2:

```
myArray=rand(1,5);
randomNumbers=10*myArray;
```

For a nested loop consisting of one loop with *m* iterations and one loop with *n* iterations, you can use a 2-dimensional array of length $m \times n$. In your case, you will want a 50×1000 array. Think carefully about what the entries in your array should be. Once you have figured that out, the other operations are similar to what you have done in the first part of the exercise.

Hint 4a

Use `help tic` for help. If you add a tic command at the start of a block of code and a toc command at the end, MATLAB will compute the time it takes to execute the block of code in between. The output will contain a line similar to this:

Elapsed time is 10.360045 seconds.

Hint 5

Remember you can use `help` if you are interested in a specific function and `lookfor` if you are interested in a specific topic (e.g. the binomial). The functions `binopdf` and `binocdf` will be of interest to you.

Hint 6

Refer to footnote 2 for explanations of pdf and cdf. You can also use the help function to find out more.

Revisions:
RTB wrote it, 23 April 2011 as "binodemo.m"
RTB revised it, 23 May 2011 as "binorat.m"
DAR updated it, 4 August 2011 as two files. This pdf and corresponding "binorat.m"
JW updated and reformatted on 8 August 2011
RTB added exercise 10 (number picking), 27 May 2012, saved as "BinoRat.m"
MIS updated, added link to hints on course website, removed number picking part to go into new file, 5 August 2013
MIS added hints at the end of the file, 15 August 2013

Rattus Binomialis - Exercise Details

Context and Aims

Day 3 of our course is dedicated to using students' programming skills to begin to think about probability and statistics. By this time, students have learned the basics of MATLAB programming, and they are now asked to apply these skills to data analysis. Our approach attempts to give students a deep, intuitive understanding of statistical principles by using computational simulations and re-sampling methods [?] with data from biological experiments.

This simulation-based approach is useful in building students' intuition about statistical concepts. It has the further advantage of requiring very little prior knowledge about existing statistical tests and methods. In addition, the general approach can be used even when conventional methods are difficult or impossible, and is therefore particularly useful in biological research.

An example of this approach is the exercise we call "Rattus binomialis," in which students calculate a p-value from the binomial distribution using a Monte Carlo simulation. The exercise is designed to simultaneously reinforce a number of key programming concepts (for-loops, indexing into arrays) and to introduce important statistical concepts (distributions, the Null Hypothesis, p-values).

Question and Presentation of the Exercise

The exercise is introduced by the instructor showing the students a movie of a rat performing a 2-alternative, forced-choice task in which it must identify an odor presented at a central port. If it detects odor "A" it should choose the right-hand port for a reward; if it detects odor "B" is should choose the left-hand port [?]. The students are then asked how they would determine whether the rat "understood" the task or whether it was guessing. A brief discussion ensues, first in small groups, then with the entire class during which it becomes apparent that they need to measure the rat's performance on a number of trials and compare this to what would be expected by chance, i.e. if the rat were just guessing. This introduces the important concept of a null model and focuses the class on developing a test that will compare the rat's behavior to this null model.

After this discussion, the instructor presents data from an experiment in which the rat performs 50 trials of the task and gets the correct answer on 31 trials (19 trials wrong) for an average of 62 % correct. The question students are asked is: What is the probability that the rat performs this well (or better) if it is only guessing?

Students are guided through a sequence of steps to complete the exercise and answer the question. First, students are asked, using the Learning Catalytics software [?], whether they think the rat is guessing. They then work in small groups of three or four students to think about how they would go about quantitatively determining an answer to this question. We tell students to use no prior knowledge they may have of probability theory, and to instead use the brute force power of a simple simulation (i.e. a random number generator and for-loops). The general approach is to simulate the experiment under the Null Hypothesis, repeat the simulated experiment many times and then ask how often the simulated result is as, or more, extreme than the value actually obtained, in order to determine a p-value. This approach is applicable to a vast variety of problems, even ones that are analytically intractable. In order to separate the task of conceptualizing the problem from the task of writing code, we ask students to start by writing "pseudocode," a succession of comment lines that break the process down into logical steps. This focuses them first on the concept of what needs to get done; then the elements of code that are needed; and finally, at a later stage, the actual code.

After discussing in small groups for about 15 minutes, attention is redirected to the instructor and a question is posed via Learning Catalytics: Write a single line of MATLAB code that will simulate a single trial under the Null Hypothesis (H_0). The code should produce a "1" if the rat guesses the correct port and a "0" if not. Since the students have had experience with the *rand* function during previous exercises,

this step usually does not take very long and many arrive at a correct solution immediately. If we find that many students get the wrong answer, we have them discuss their answers in small groups and we then re-pose the question. Invariably this think-pair-share exercise results in everyone getting the correct answer. We next ask them to prove to themselves that their line of code works by running it several thousand times and plotting a histogram to see if they get the expected 50:50 distribution of 1s and 0s.

Students then proceed to simulate one experiment by running 50 trials with a for-loop and storing the results (the number of times the rat guesses correctly) in a variable. They then simulate the running of 1000 such experiments by embedding the "experiment" for-loop inside a second "simulation" for-loop. The students then tally up how often in these 1000 simulated experiments the rat produced 31 or more correct guesses and divide this tally by the number of simulations to determine how likely it is to obtain the observed result under H_0. Students with strong prior training in statistics might recognize that this number is, in fact, the p-value, but this is not, at this point, made explicit. The procedure itself and the rationale behind it are accessible even to students with a limited statistical background.

After about 15 minutes, most students have come up with a script that compiles and generates a p-value, and we ask them to submit their answer via Learning Catalytics. Often the number is wildly off, because the students have forgotten to re-initialize their storage array between simulations or some other coding mistake. The de-bugging is carried out by a combination of roving TAs and by encouraging students to compare their answers and code with each other. As a final step, the instructor types in a script while encouraging input and/or responses from the class (by asking questions such as "Where should I initialize the variable that is holding single-trial results?" or "What does this line of code do?")

The next step of the exercise serves to make an explicit connection between the exercise students have just completed and the concept of a p-value. The instructor poses the following question via Learning Catalytics: "What is a p-value?" The responses often reveal common misconceptions, for instance, that the p-value is "the probability of wrongly rejecting the Null Hypothesis" [**?**], or that the p-value indicates the probability of the Null Hypothesis being true given the data [**?**]. At this point, the instructor asks the students to go back and look at their code and the p-value they actually calculated and then to re-answer the question. The instructor might need to clarify the issue by recapitulating the process: "We assumed that the Null Hypothesis was true, generated a distribution of number of correct trials out of 50, and then we asked how likely it was to get the answer we actually got (31 correct) or one more extreme (> 31 correct). This is how a p-value is obtained." This helps students understand the nature of a p-value.

For students who solve the problem quickly, we introduce a number of extensions, such as writing code with the same functionality but without using for-loops or converting their MATLAB script to a function that can accept any value for "percent correct" and number of trials and return the p-value. The complete "Rattus binomialis" module typically requires 1.5 to 2 hours for our first-year graduate students.

A document that guides students through the sequence of steps and that offers hints to the students can be found on the course github repository: `https://github.com/MelanieIStefan/QMBC`. MATLAB code for a sample solution is provided in supporting file S4. The sample solution is provided to students at the end of the day.

Student learning

The "Rattus Binomialis" exercise is part of an entire course day devoted to statistics. The module teaches and reinforces ideas and techniques around using computer simulations for hypothesis testing. In addition, students are asked to use simulations to build intuition about random processes and statistical concepts.

Students with previous exposure to statistical concepts such as p-values or the Binomial distribution have an opportunity to "re-discover" the meaning of these concepts through simulations, and thus reinforce them. For students with a limited statistics background, these concepts will be more difficult, and might not be immediately understood. Even so, the "Rattus binomialis" exercise is valuable, because it introduces everyone to the idea of simulation-based statistics. It also reinforces the programming skills students learned during the first two days of the course.

In the basic module, students are not explicitly asked to visualize and summarize their data, but they might choose to do so, both to support their problem-solving and to validate their thinking. An overview of the Day 3 learning goals covered in this module is given in table 1. In addition, the exercise relies on programming skills and concepts introduced during the first two days, thereby reinforcing them.

Table 1. Statistics learning objectives addressed in the Rattus Binomialis exercise

Visualize and summarize data
- Plot experimental data and outcomes of simulations<br>- Compute summary statistics from a dataset
**Use simulations to build intuition about statistical concepts**
**- Use computer simulations to build intuition about random variables**<br>**- Simulate different kinds of random distributions**<br>- Simulate sampling distributions<br>- Use computer simulations to inform experimental design
**Use simulations for hypothesis testing**
**- Articulate and encode the Null Hypothesis**<br>**- Generate a simulated distribution under the Null Hypothesis**<br>**- Compare simulated results to data and obtain a p value**<br>**- Accept or reject a hypothesis based on the outcome of a simulation experiment**<br>**- Compare simulation-based approaches to standard hypothesis tests**

Statistics learning objectives addressed in the Rattus Binomialis exercise. All learning objectives for Day 3 are shown, with those reinforced in the Rattus Binomialis exercise shown in bold.

The exercise also reinforces the three big themes of the course: Students are presented with a problem and asked to solve it systematically by breaking it down into manageable parts. They need to find a way of implementing their solution, and they are encouraged to experiment with code and find ways of testing their implementation and comparing it to other possible solutions.

Learning can be understood as a process of moving towards more sophisticated levels of understanding. For instance, mere recall of a number of facts is a relatively low-level form of learning, while the synthesis of those facts to obtain a deeper understanding constitutes a higher level. This "taxonomy" of levels of understanding has first been proposed by Bloom [?]. Bloom's taxonomy has since been modified to a version applicable to computing subjects [?]. The revised taxonomy accounts for the observation that in computer science, theoretical understanding does not always progress at the same rate as the ability to create code. It therefore understands learning as a progression along two dimensions: towards the right on the "interpretation" dimension and towards the top on the "creation" dimension [?].

If we apply this idea to the Rattus Binomialis exercise, we can frame each step of the problem in terms of the learning experience.

For instance, in step 1, students are asked to simulate a single trial under the Null Hypothesis, that is, under the assumption that the rat is guessing. Students solve this problem by understanding that this amounts, essentially, to a coin toss, and can then apply what they have learned about random functions to implement a piece of code that achieves the required result. Their initial solution is thus likely to be in the Apply/Understand quadrant of the two-dimensional taxonomy. At this point, we challenge students to prove to themselves that their solution is, indeed, correct. This involves producing a new piece of testing code that will repeat the experiment many times, and checking that this does indeed reproduce the behavior of a fair coin flipped repeatedly. This moves students to the right and up in the table, into the "Create/Evaluate" quadrant, and thereby to a more sophisticated level of mastery. Two possible

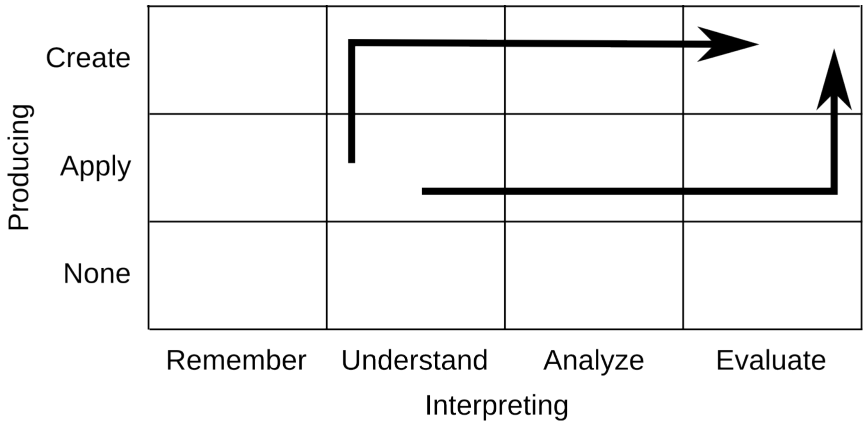

Figure 1. Possible paths through the Fuller-Bloom taxonomy [?] for step 1 of the Rattus Binomialis exercise.

trajectories for this step are visualized in figure 1. The repeated simulation of the same event also helps students build intuition about the law of large numbers: With large numbers of repetitions of a (fair) coin flip, the proportion of times the coin lands on "heads" will get closer and closer to 50 %.

As students move through the entire exercise, the focus shifts back and forth between creating (producing code) and understanding (e.g. when statistical concepts are discussed in class). Advanced students receive additional challenges in both dimensions by extra steps that require them to create new implementations (e.g. making the code more efficient by replacing the double for-loop with a vectorized solution, step 4) and/or to extend their understanding (e.g. exploring distributions other than the binomial distribution, step 8). Thus, the "Rattus binomialis" module asks students to develop both their code production skills and their understanding, and caters to beginners as well as more advanced students.

Follow-up exercises

The simulation-based approached to probability and statistics is further extended and reinforced by an in-class exercise to conduct a two-sample test of real neuroanatomical data, and in two homework exercises. In the first homework exercise, students are presented with data from a previous class where each student was asked to pick an integer between 1 and 4 (inclusive). If every student picked a number at random, one would expect a roughly similar number of students to have picked each number. In the dataset at hand, however, the number 3 is overrepresented. Indeed, at every session of the Quantitative Methods Boot Camp, we ask students to pick a number between 1 and 4 on the first day of class, and every time, the number 3 is picked most often. Do people in the class have a bias toward the number 3, or is this result within the range of outcomes if everyone truly picks at random? The exercise is similar in structure to the "Rattus binomialis" exercise and can be solved using a similar simulation-based approach. The formulation of the Null Hypothesis takes a little bit more thought and leaves room for individual interpretation. For instance, H_0 could be "The number 3 is not picked with a higher frequency than other numbers," or it could be "No single number is picked with a higher frequency than other numbers." Students have to think carefully about the way they frame the question and the way they document and justify their choices.

Another homework exercise takes students through the "Birthday problem." This problem explores the probability that two (or more) students in one class share the same birthday. Contrary to most people's intuition, this probability reaches 50 % as soon as the class has 23 students. In courses about probability and statistics, this problem is often solved algebraically, which involves thinking about the complementary

event (no two people share a birthday) and computing the probability of that using combinatorics. In this class, students do not need to do that, or even know that this is possible: They can solve the problem by simulating 10000 classes of n people each, and count the number of times two or more of them share a birthday.

These two homework exercises again showcase the ease and practicality of simulation-based approaches to statistics. They also reinforce concepts and skills in both statistics and programming.
