# Supplementary material for: The Quantitative Methods Boot Camp: Teaching Quantitative Thinking and Computing Skills to Graduate Students in the Life Sciences
Source: PLoS Comput Biol. 2015 Apr 16;11(4):e1004208. doi: 10.1371/journal.pcbi.1004208 (PMC4399943; doi:10.1371/journal.pcbi.1004208)
Supplement: S3 Text — (PDF) [file pcbi.1004208.s004.pdf]

# Quantitative Methods Boot Camp (QMBC): Assessment Supplement

MI Stefan, JL Gutlerner, RT Born, M Springer

## Contents

**1 Day 5 - bring your own research/special topic day** **2**

**2 Course assessment and data presentation** **3**

**3 Rating of specific course components** **4**

**4 Self-assessed programming skills** **8**

**5 Students self-assessment on key skills** **14**

**6 Projected long-term impact** **22**

# 1 Day 5 - bring your own research/special topic day

On the last day of the course, students have a chance to work on their own research or topic of interest. There are several key factors we find are useful to making this approach successful: 1) Most types of research problems and data sets fall into a few general categories, so it is easy to group students by topics of interest to them. 2) We separate the class by topic area, allowing them to focus on an area that interests them and allowing them to collaborate with colleagues with similar interest. 3) We encourage students to work with a peer, which facilitates problem solving. 4) We have a high TA to student ratio, allowing students to receive ample individual attention.

Near the end of day 4, we query the students by Learning Catalytics to get a short description of what they intend to do on day 5. This lets us plan how to divide the class into groups on day 5. Often the class breaks into several large groups, e.g. bioinformatics or image analysis, and several smaller groups, e.g. specific data parsing issues. Each group is assigned a TA (or instructor) with expertise in the group's topic. Groups larger than 10 people are subdivided either arbitrarily or based on cursory similarity of research problem. Small groups are sometimes combined. Each individual in the group then spends one to five minutes discussing their particular research problem. As best as possible students are further paired up so they are working in small teams of very similar problems or approaches. This peer-to-peer interaction is important, as we anecdotally have found these small groups are less likely to get stuck. The rest of the time is then spent with TAs and instructors working one-on-one with individuals in the class. This sometimes can be a very quick check-in and sometime in can be a 15-30 minute discussion. During the day each group informally discusses, with others in their larger group, the approaches they have taken to their problem, the successes, and challenges; sometimes this leads to good suggestions or discussions. Given the high TA to student ratio, each student spends an average of one hour working directly with a TA during the day. Upper division students often comment that this is the most useful day of the course.

# 2 Course assessment and data presentation

## Delivery

Students were asked to fill out an anonymous electronic survey at the conclusion of each iteration of QMBC.

## Number of respondents

Total numbers of respondents were as follows: 37 (50 % of students enrolled) in Spring 2012, 21 (57 %) in Spring 2013, 24 (55 %) in Spring 2014, 57 (61 %) in Summer 2012, and 43 (58 %) in Summer 2013.

## Presentation of qualitative data

Most questions involved a five-point Likert scale, where students had to rate either the quality of some aspect of the course (as "Poor", "Fair", "Good", "Very Good", or "Excellent"), their agreement with a statement ("Strongly Disagree" to "Strongly Agree"), or their likelihood of doing something ("Definitely Will Not" to "Definitely Will"). All Likert data is represented as diverging stacked bar charts. Every stack has the same total length, representing 100 % of the students who responded to a question. Colored segments are proportional in length to the number of students who gave a specific answer. For quality ratings, we use a sequential color scheme (darkest for "Poor", lightest for "Excellent"), and center the stacked bar charts at the border between "Fair" and "Good" (representing the neutral point in our data). For ratings of agreement, we use a different color scheme (darkest for strong agreement or disagreement, lightest for neutral; going from dark blue for "Strongly Disagree" to dark red for "Strongly Agree") and center the stacked bar graph in the middle of the neutral category ("Neither Agree nor Disagree"). The representation of likelihood data is analogous.

## Quantitative analysis

We show and report what proportion of students gave a particular answer or a set of answers. Because of the small number of course offerings so far and the relatively small number of students in each class, we do not try to draw strong statistical conclusions.

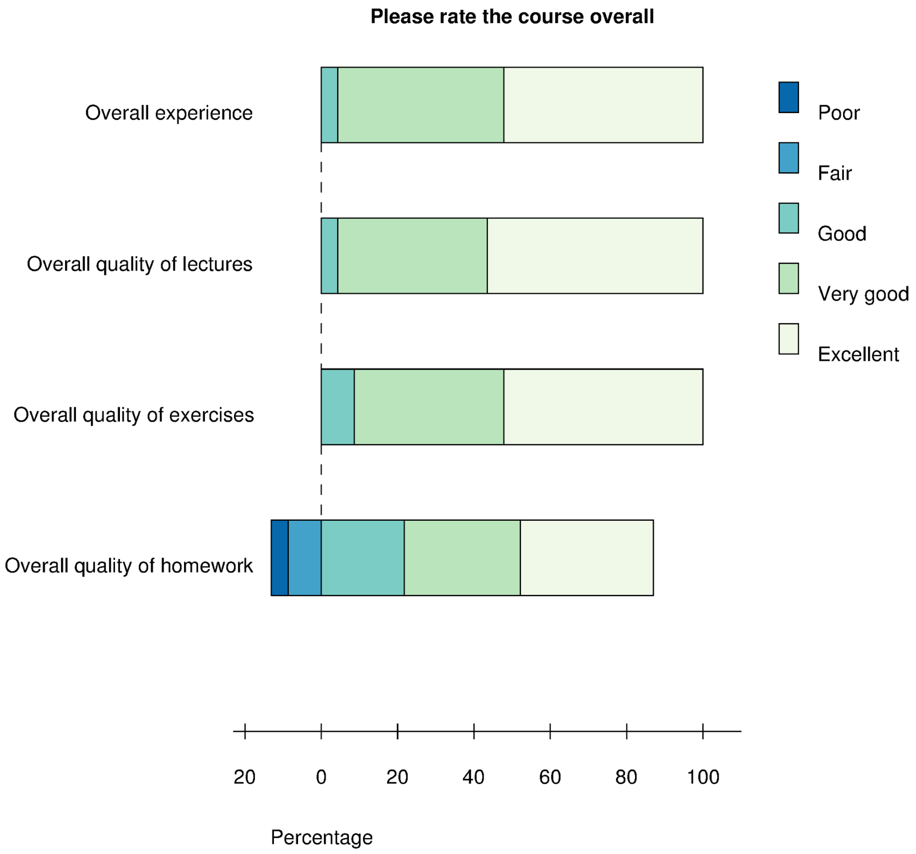


Figure S1: **Student satisfaction with lectures, exercises and homework in the Spring 2014 offering of Boot Camp.** Students rated their experience on a five-point scale (Poor to Excellent, as above). $n = 24$

# 3 Rating of specific course components

In order to explore areas with further potential for improvement, we investigated students' satisfaction with the course in more detail by asking them how they ranked aspects of the course (lectures, in-class exercises and homework). Figure 1 below shows the results for the most recent offering of the course (Spring 2014). Students' ratings of aspects of the course in previous years are shown in figure 2 (lectures), figure 3 (exercises), and figure 4 (homework).

In the Spring 2014 offering, students show great satisfaction with the lectures and in-class exercises, with all students rating them as "good", "very good" or "excellent". The quality of homework exercises received slightly lower ratings (87 % of ratings "good" or better). Revising and improving homework questions is therefore a key priority for future iterations of the course. Thus, our surveys

have helped us to identify areas of in need of development in order to continually improve the course.

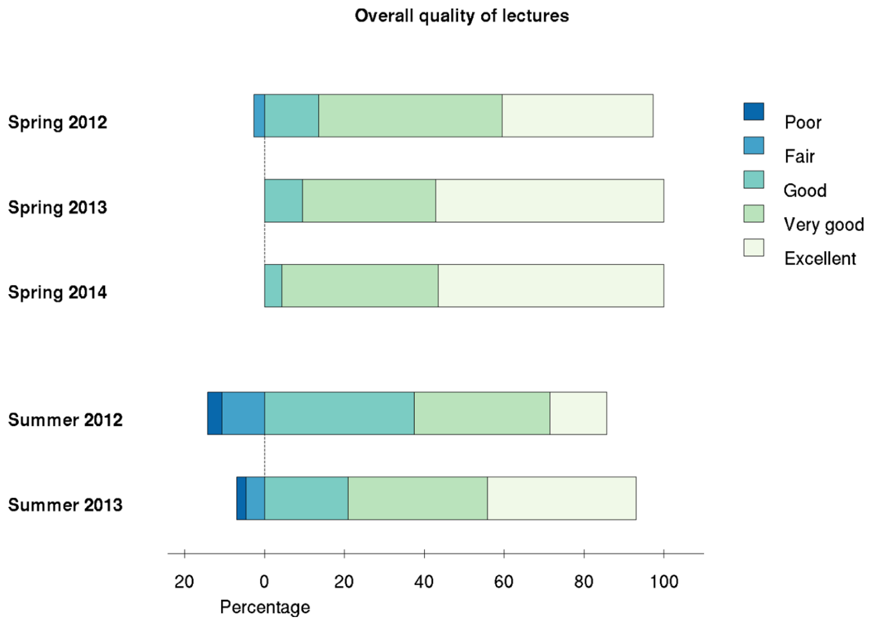


Figure S2: **Students' rating of overall lecture quality, 2012-2014.**

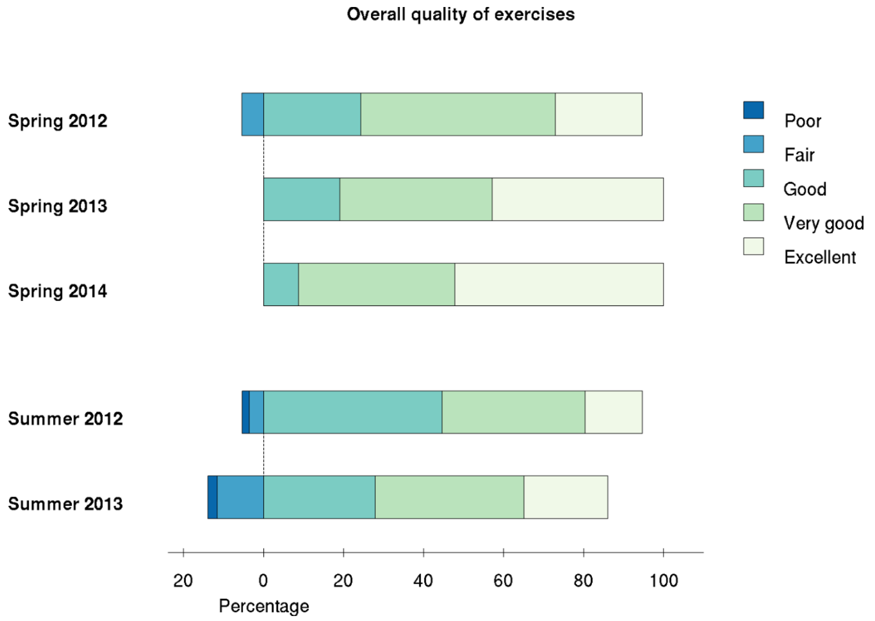


Figure S3: **Students' rating of course exercises, 2012-2014.**

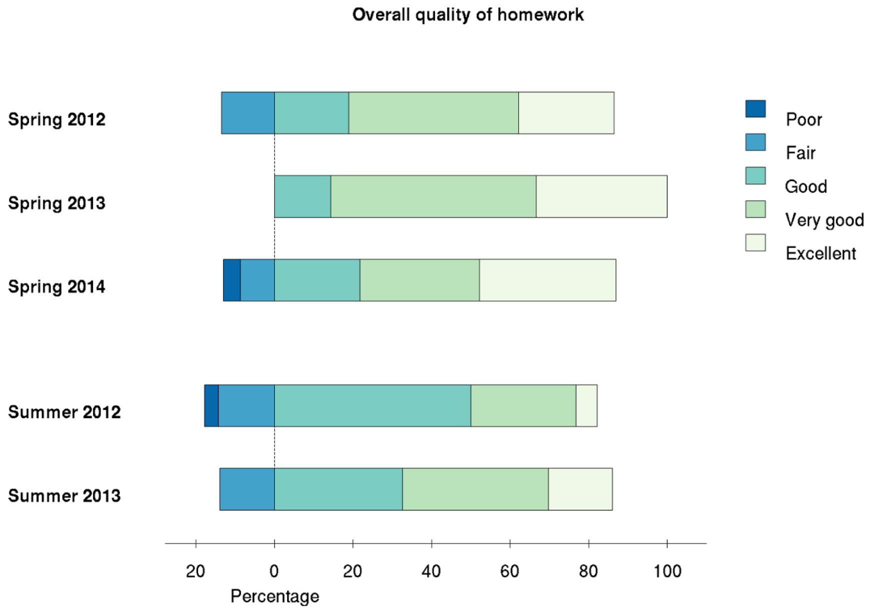


Figure S4: **Students' rating of homework, 2012-2014.**

## 4 Self-assessed programming skills

### MATLAB

Figure 2 of the paper shows students' rating of their MATLAB programming skills on a 0 to 10 scale (novice to expert) before and after the course, as reported in the end-of-course survey.

In each class, the median improvement in skill level was 3 points on an eleven-point scale. While almost all students reported improvement in their skills, learning gains tend to be highest for students who came in as beginners or relative beginners. This might reflect a ceiling effect, but is also in line with the design of the course as a low-threshold introduction to quantitative methods for absolute beginners. The few students who report no increase in their skills belong exclusively to the group of incoming graduate students who are already highly skilled in this area. This indicates that there is room for improvement when catering to the needs of advanced students. Together, the results indicate that the vast majority of our students feel like they have learned something from the course, with self-reported learning gains being highest among students with little or no background knowledge.

### Programming in general

A survey of general programming skills (not necessarily MATLAB) showed similar results. Data from Spring 2012 to Spring 2014 is shown in figures 5 to 9, respectively.

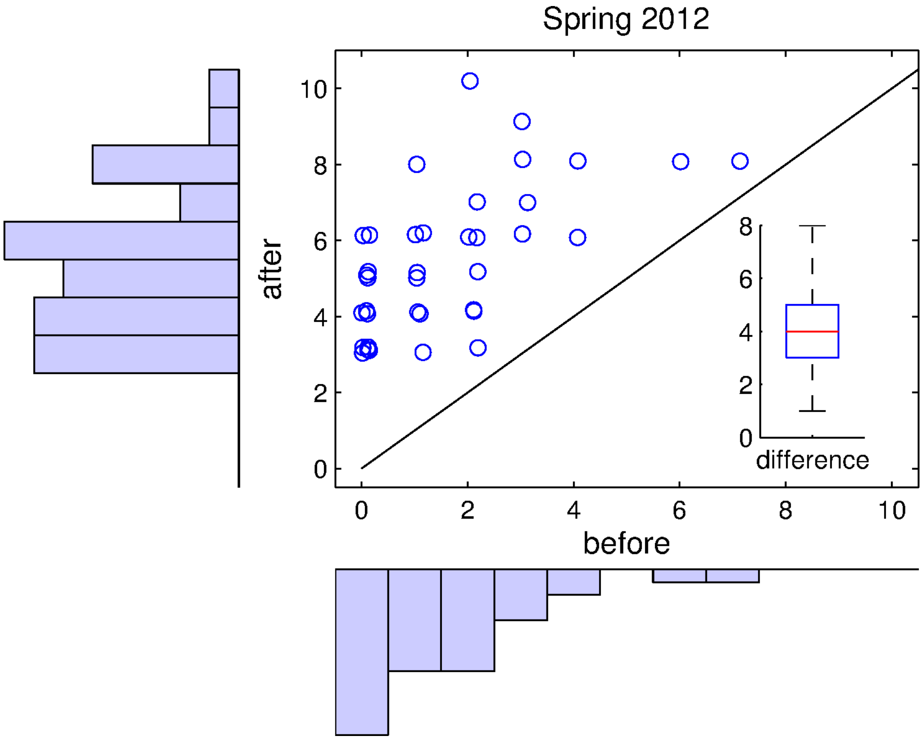


Figure S5: **Increase in students' understanding of the course material (Spring 2012).** Students were asked: "Rate your understanding of the material before and after the course on a scale form 0-10 (0=none; 10=expert)"

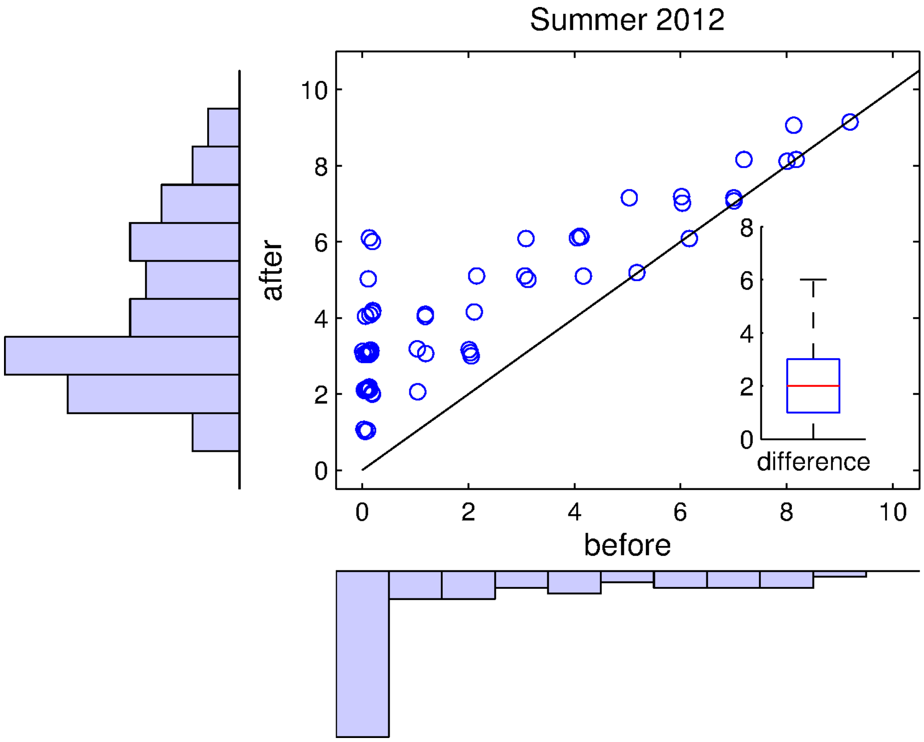


Figure S6: **Increase in students' programming skills (Summer 2012).** Students were asked: "Rate your ability to program: (0 = novice; 10 = expert)"

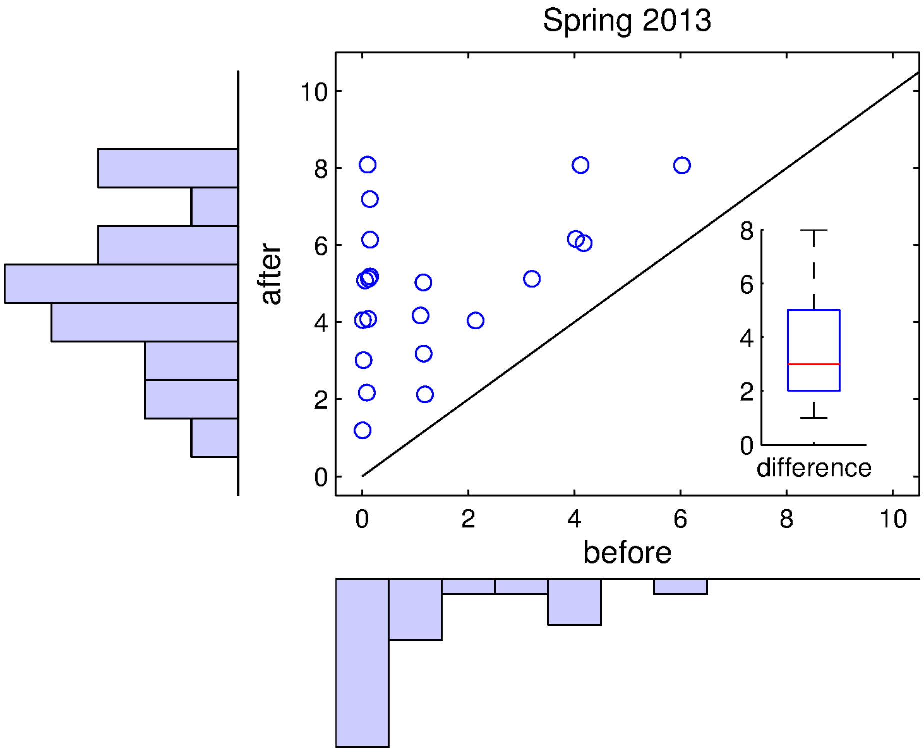


Figure S7: **Increase in students' programming skills (Spring 2013).** Students were asked: "Rate your ability to program: (0 = novice; 10 = expert)"

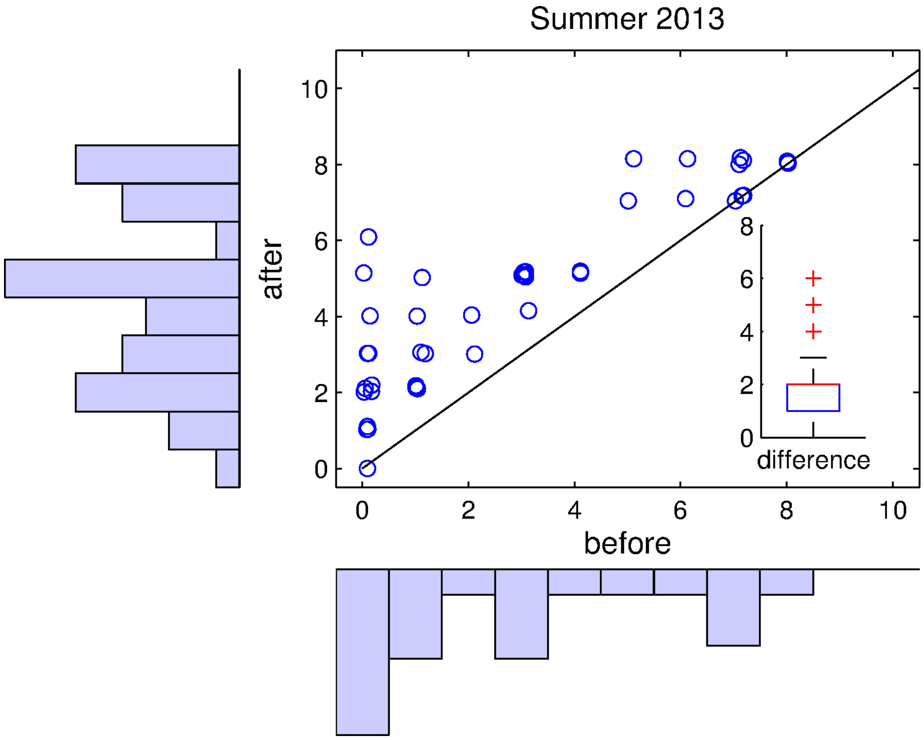


Figure S8: **Increase in students' general programming skills (Summer 2013).** Students were asked: "Rate your ability to program in any language: (0 = novice; 10 = expert)"

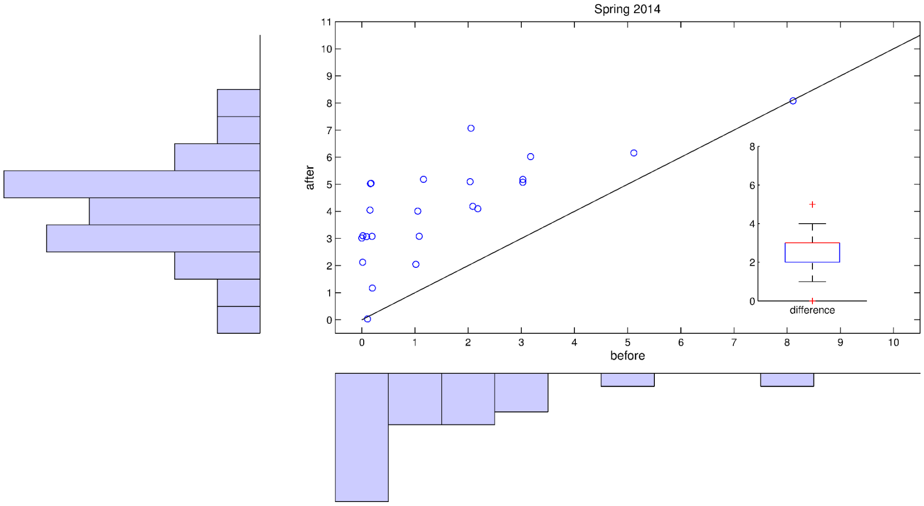


Figure S9: **Increase in students' general programming skills (Spring 2014).** Students were asked: "Rate your ability to program in any language: (0 = novice; 10 = expert)"

## 5 Students self-assessment on key skills

In order to assess how well students understood specific daily learning objectives, we ask them to rate their understanding of a few of the key concepts on a five-point scale. Students' ratings for the spring 2014 offering are shown in figure 3 of the main text.

Students report a high understanding of concepts covered early in the course, such as arrays (100 % reporting an understanding of "good" or better) and loops (100 %). This reflects the fact that those concepts are continually applied and reinforced during later parts of the course. Students find concepts from the later days of the course more difficult, especially those relating to more complex aspects of statistics, such as articulating a Null Hypothesis and calculating a p-value. Fifty-seven percent of students report a good or better understanding of how to formulate a Null Hypothesis, while 48 % report a good or better understanding of how to interpret a p-value. We do not think these values represent a failure, since these concepts require sophisticated thinking about statistics, and misconceptions about them have been documented even among scholars and researchers in biomedical fields[**?**, **?**]. We also do not have pre-course ratings against which to compare them. Nevertheless, it is clear that a single day of instruction is not enough to explore statistical concepts in the same depth as a semester-long course would. Rather, our goal is to give students an appreciation of the power of simulation-based approaches to statistics, and to teach them how to implement them.

Students' self-reported ratings of specific skills and concepts for the last five offerings of the course are shown in figures 10 to 19. In general, students in the spring offerings of our course rate their skills higher upon completion of Boot Camp than students in the summer offerings. Again, this probably reflects the higher student motivation and smaller class sizes of our spring cohorts. For both types of classes, we see a trend towards higher reported skill levels over the years.

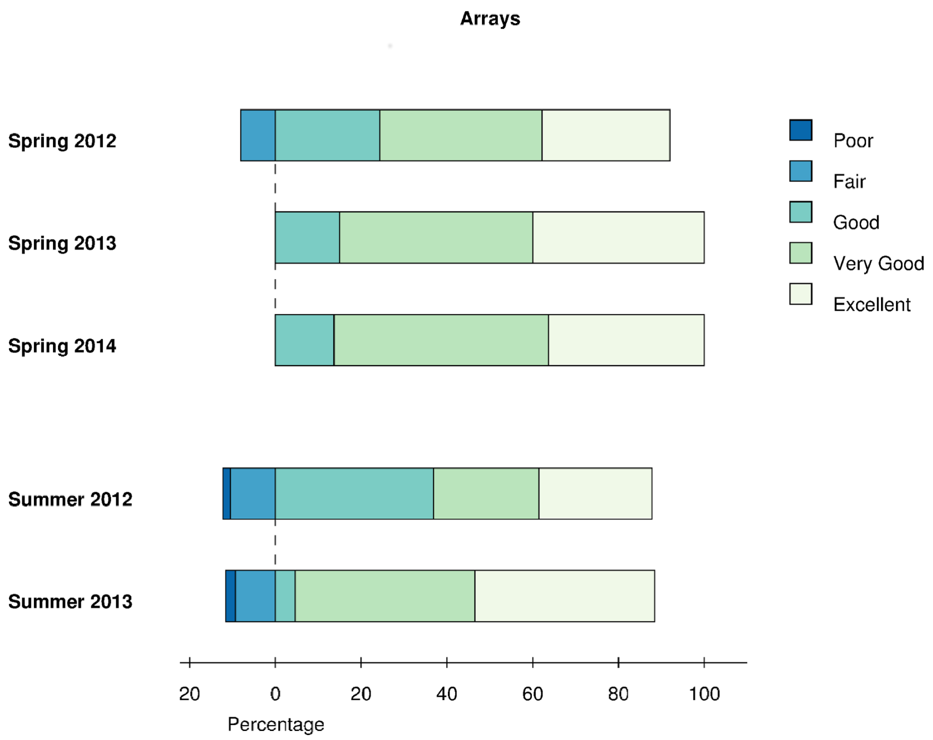


Figure S10: **Students' understanding of arrays.** Students were asked to rate their own understanding at the end of the course. Data is shown for all offerings of the course between 2012 and 2014.

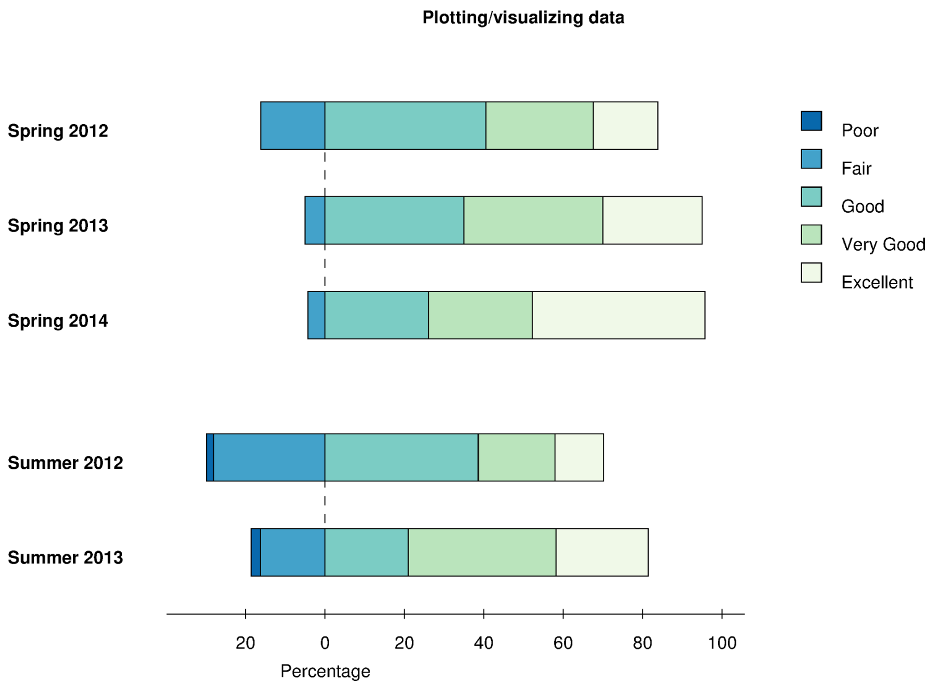


Figure S11: **Students' understanding of data visualization.**

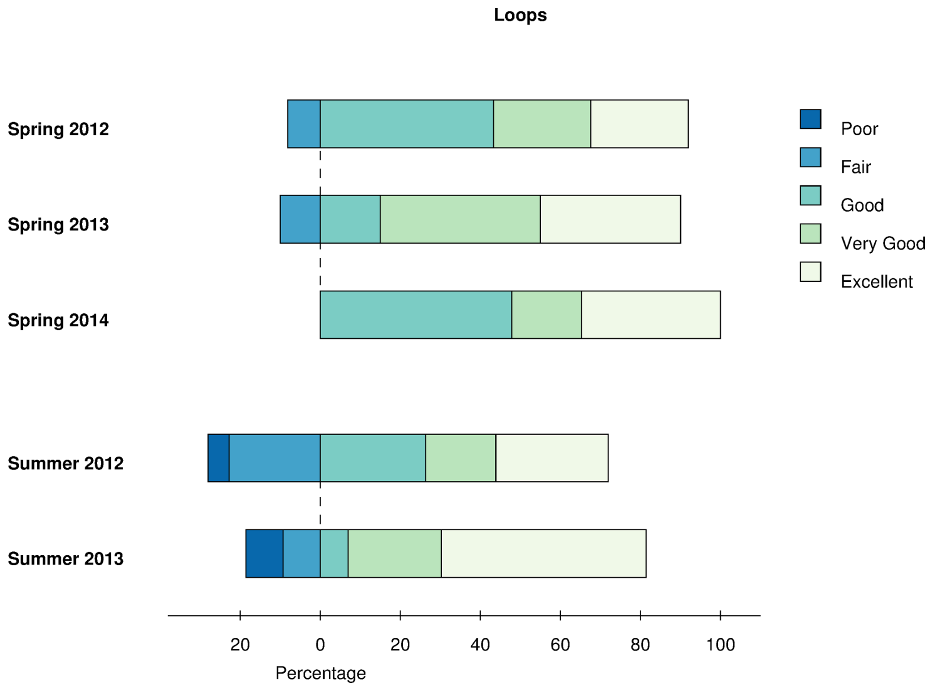


Figure S12: **Students' understanding of loops.**

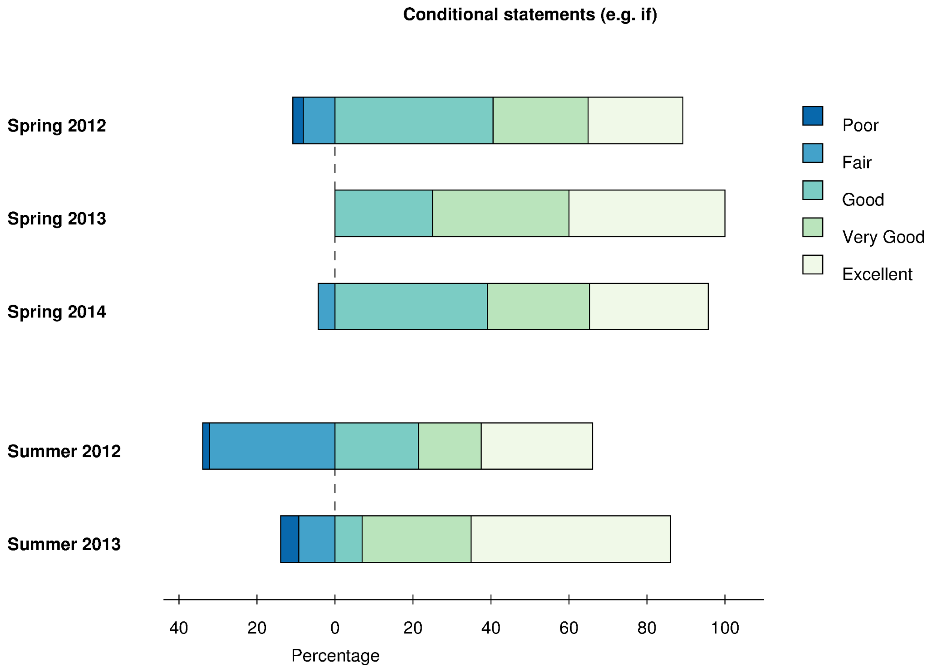


Figure S13: **Students' understanding of conditional statements.**

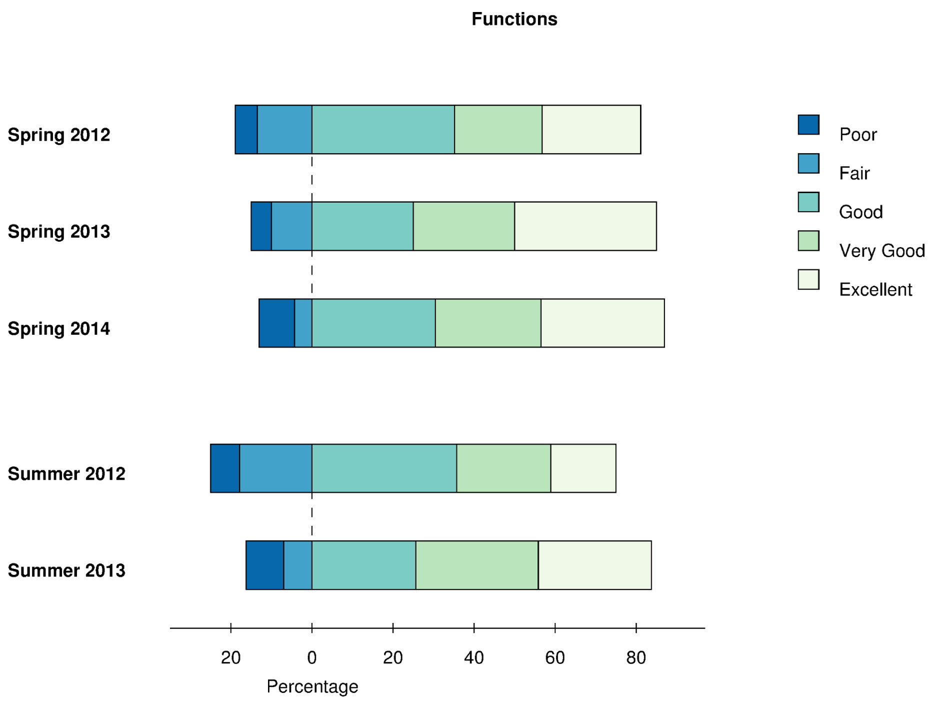


Figure S14: **Students' understanding of functions.**

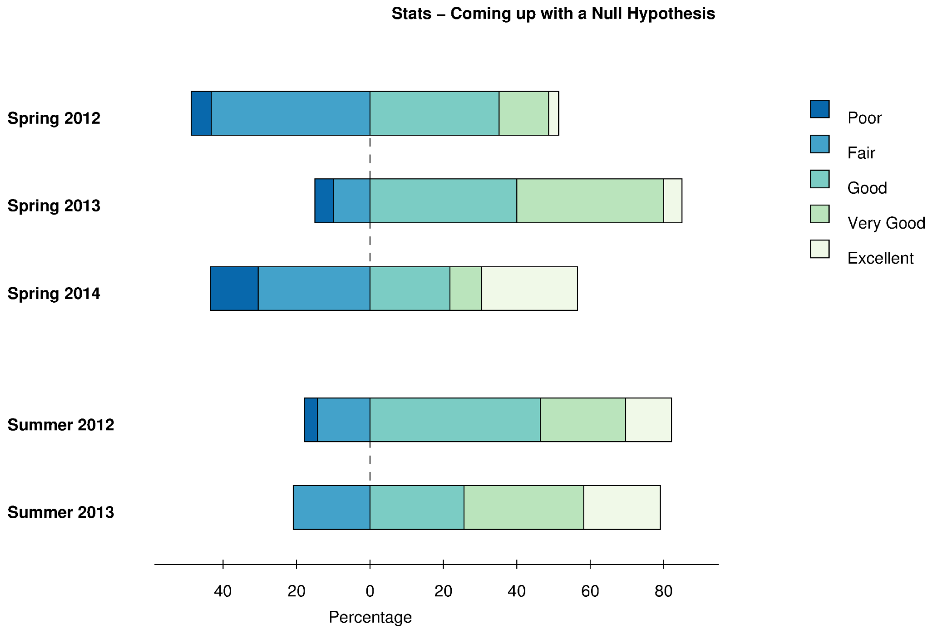


Figure S15: **Students' understanding of formulating a Null Hypothesis.**

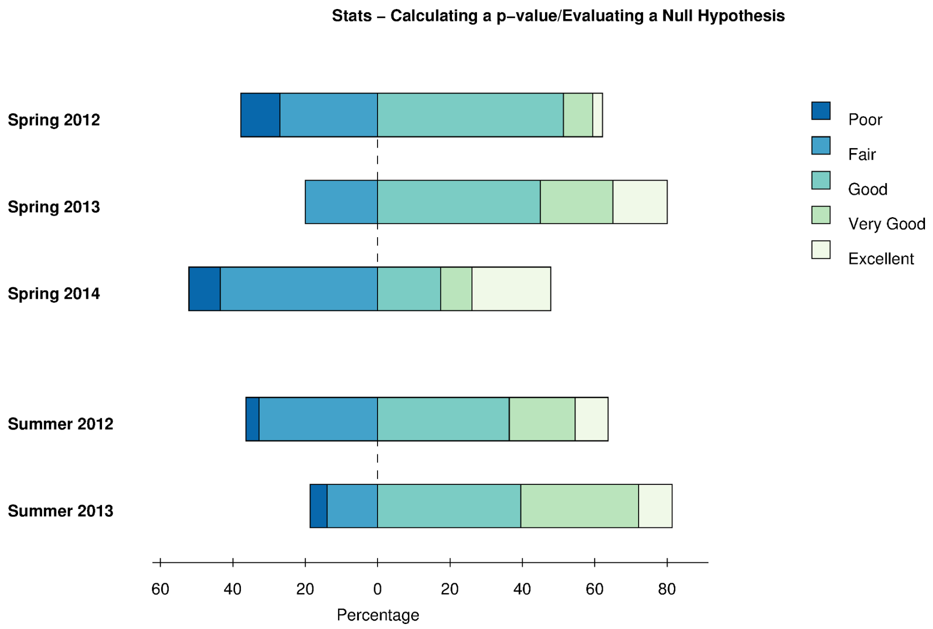


Figure S16: **Students' understanding of interpreting a p-value / evaluating a Null Hypothesis.**

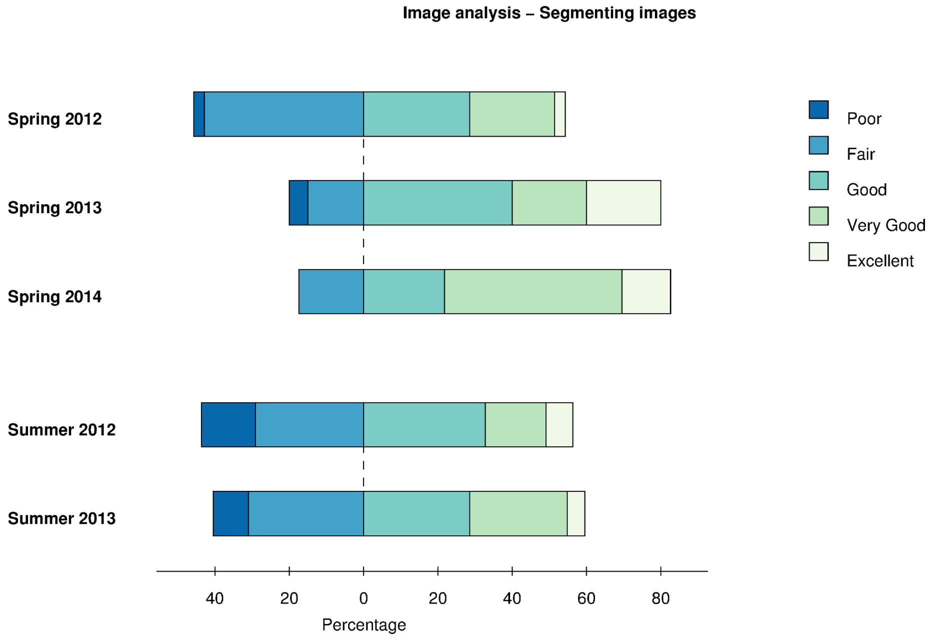


Figure S17: **Students' understanding of image segmentation.**

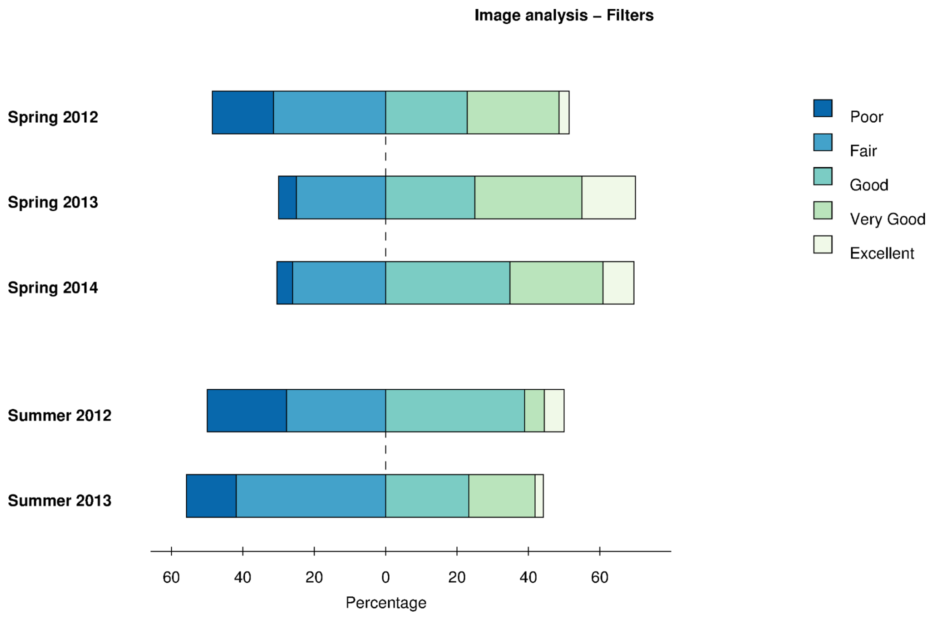


Figure S18: **Students' understanding of image filtering.**

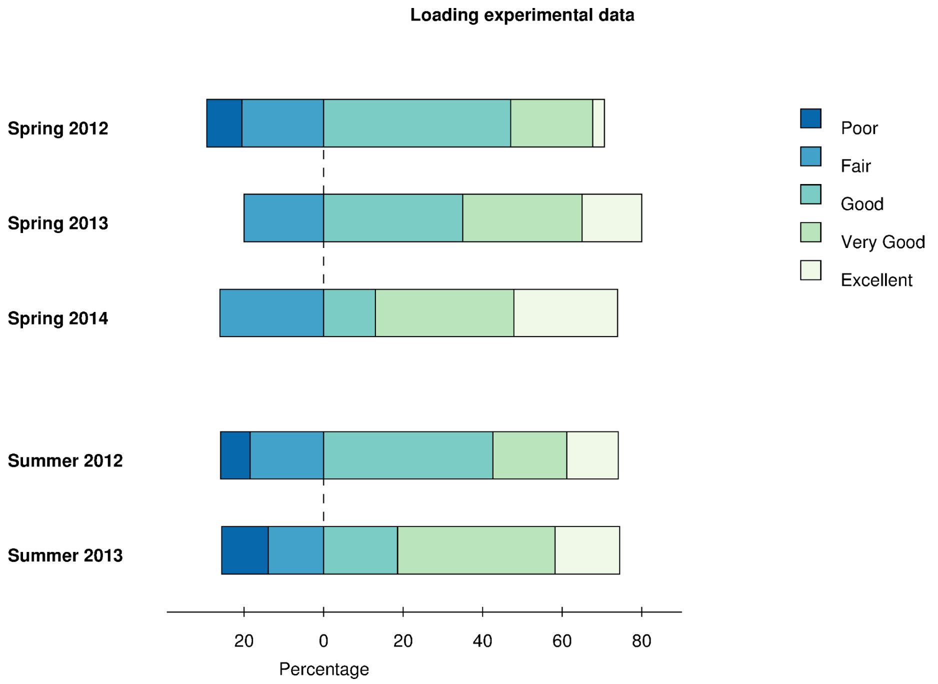


Figure S19: **Students' understanding of data loading.**

# 6 Projected long-term impact

To assess the potential long-term impact of the course, we surveyed students to rate their preparedness for quantitative problem-solving, working with MATLAB and using quantitative methods in their research on a five-point scale. Pooled data from the last four offerings of the course (Summer 2012 to Spring 2014) is shown in figure 4 of the main text. (In the Spring 2012 course survey, the questions were not included in the same format.) The total number of survey respondents was 141.

One of our learning goals was to instill habits of quantitative thinking and problem-solving in our students. To see whether this goal is achieved, we asked students in the post-course survey to rate their agreement with the statement "The workshop provided me with a practical base/starting point for analyzing quantitative problems."

As is evident in figure 4 of the paper, most (89 %) of our students "agree" or "strongly agree" that the course has provided them with a starting point for analyzing quantitative problems. This reflects positively on students' learning gains in the cognitive domain. It also reflects positive outcomes in the affective domain, notably that students recognize the value of quantitative approaches and feel confident about using them.

In the skills domain, our aim was to equip students with the practical skills to get started on solving problems with MATLAB. As reported above, most students reported an increase in their MATLAB programming skills. We also wanted to know whether they were likely to use MATLAB in the future. We therefore asked them to rate their agreement with the statement "This course provided a practical base and starting point for using MATLAB in my own work." Again, figure 4 of the paper shows that the overwhelming majority (88 %) of our students agreed or strongly agreed with this statement.

Third, we were interested in knowing whether the course reinforces a positive attitude towards quantitative thinking and the use of computational tools among our students. We therefore asked students to indicate their agreement with the statement "This course has increased the likelihood I will use quantitative methods in my research." Most students (78 %) did indeed either "agree" or "strongly agree" with this statement.

Finally, we wanted to know whether students were likely to spread their enthusiasm for the subject matter. We asked students in the post-course survey "Would you recommend this course to future students?" Responses to this question are shown in figure 20 below. Every year, more than 85 % reported that they would probably or would definitely recommend the course to future students.

This is a further indication that students enjoy the course. But it is also a sign that they are willing to convince others of the benefits of computational skills and quantitative thinking. This speaks to our learning goal of influencing others and spreading good quantitative and computational practices.

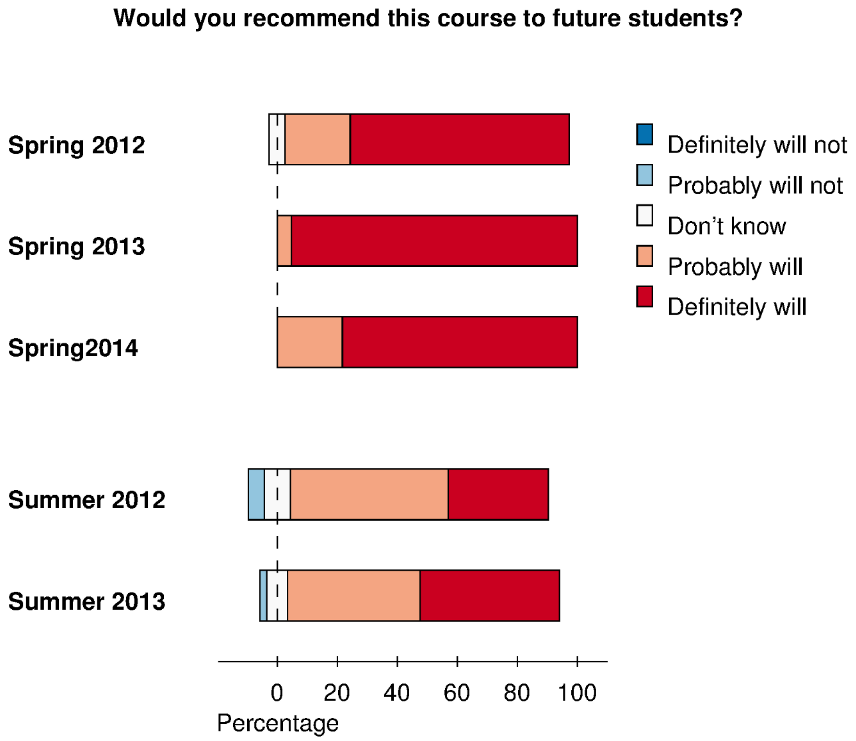


Figure S20: **Willingness to recommend the class.** Students were asked to rate whether they would recommend the class to future students on a five-point scale (Definitely will not to definitely will). Data is shown centered at Don't know. Spring 2012: $n = 37$, Spring 2013: $n = 21$, Spring 2014: $n = 24$, Summer 2012 $n = 57$Summer 2013: $n = 43$.
