# Supplementary material for: The Quantitative Methods Boot Camp: Teaching Quantitative Thinking and Computing Skills to Graduate Students in the Life Sciences
Source: PLoS Comput Biol. 2015 Apr 16;11(4):e1004208. doi: 10.1371/journal.pcbi.1004208 (PMC4399943; doi:10.1371/journal.pcbi.1004208)
Supplement: S4 Text — (PDF) [file pcbi.1004208.s005.pdf]

Uses of Learning Catalytics[1]

**Automated response system to ask questions during class**
Uses:<br>- Gauge students' understanding of concepts in real time<br>- Tailor and pace instruction to needs of the class<br>- Conduct think-pair-share exercises[2]<br>- Poll students about feelings and attitudes
**Electronic seating chart**
Uses:<br>- Record students' attendance<br>- Learn students' names more quickly<br>- Identify individual students who find a concept difficult and help them<br>- Answer individual questions
**Self-paced study modules**
Uses:<br>- Allow students to make their way through longer in-class exercises step-by-step<br>- Record answers to homework questions and provide immediate automated feedback<br>- Enable automatic grading

References

[1] Schell J, Lukoff B, Mazur E (2013) Increasing Student Engagement and Retention using Classroom Technologies: Classroom Response Systems and Mediated Discourse Technologies, Emerald, chapter Catalyzing Learner Engagement Using Cutting-Edge Classroom Response Systems in Higher Education. Cutting-edge Technologies in Higher Education. pp. 233–261. URL `http://mazur-www.harvard.edu/publications.php?function=display&rowid=701`.

[2] McTighe J, Lyman FT (1988) Cueing thinking in the classroom: The promise of theory-embedded tools. Educational Leadership 45: 18–24.
